# Dynamic quinone repertoire accompanied the diversification of energy metabolism in *Pseudomonadota*

**DOI:** 10.1093/ismejo/wrae253

**Published:** 2024-12-18

**Authors:** Sophie-Carole Chobert, Morgane Roger-Margueritat, Laura Flandrin, Safa Berraies, Christopher T Lefèvre, Ludovic Pelosi, Ivan Junier, Nelle Varoquaux, Fabien Pierrel, Sophie S Abby

**Affiliations:** Univ. Grenoble Alpes, CNRS, UMR 5525, VetAgro Sup, Grenoble INP, TIMC, Grenoble 38000, France; Univ. Grenoble Alpes, CNRS, UMR 5525, VetAgro Sup, Grenoble INP, TIMC, Grenoble 38000, France; Univ. Grenoble Alpes, CNRS, UMR 5525, VetAgro Sup, Grenoble INP, TIMC, Grenoble 38000, France; Univ. Grenoble Alpes, CNRS, UMR 5525, VetAgro Sup, Grenoble INP, TIMC, Grenoble 38000, France; Aix-Marseille Université, CNRS, CEA, Institute of Biosciences and Biotechnologies of Aix-Marseille, Saint-Paul-lez-Durance 13108, France; Univ. Grenoble Alpes, CNRS, UMR 5525, VetAgro Sup, Grenoble INP, TIMC, Grenoble 38000, France; Univ. Grenoble Alpes, CNRS, UMR 5525, VetAgro Sup, Grenoble INP, TIMC, Grenoble 38000, France; Univ. Grenoble Alpes, CNRS, UMR 5525, VetAgro Sup, Grenoble INP, TIMC, Grenoble 38000, France; Univ. Grenoble Alpes, CNRS, UMR 5525, VetAgro Sup, Grenoble INP, TIMC, Grenoble 38000, France; Univ. Grenoble Alpes, CNRS, UMR 5525, VetAgro Sup, Grenoble INP, TIMC, Grenoble 38000, France

**Keywords:** proteobacteria, ubiquinone, menaquinone, rhodoquinone, biosynthetic pathway, energy metabolism, adaptation, respiration, fumarate and nitrate reductase regulator- fnr, oxygen

## Abstract

It is currently unclear how *Pseudomonadota*, a phylum that originated around the time of the Great Oxidation Event, became one of the most abundant and diverse bacterial phyla on Earth, with metabolically versatile members colonizing a wide range of environments with different O_2_ concentrations. Here, we address this question by studying isoprenoid quinones, which are central components of energy metabolism covering a wide range of redox potentials. We demonstrate that a dynamic repertoire of quinone biosynthetic pathways accompanied the diversification of *Pseudomonadota*. The low potential menaquinone (MK) was lost in an ancestor of *Pseudomonadota* while the high potential ubiquinone (UQ) emerged. We show that the O_2_-dependent and O_2_-independent UQ pathways were both present in the last common ancestor of *Pseudomonadota*, and transmitted vertically. The O_2_-independent pathway has a conserved genetic organization and displays signs of positive regulation by the master regulator “fumarate and nitrate reductase” (FNR), suggesting a conserved role for UQ in anaerobiosis across *Pseudomonadota*. The O_2_-independent pathway was lost in some lineages but maintained in others, where it favoured a secondary reacquisition of low potential quinones (MK or rhodoquinone), which promoted diversification towards aerobic facultative and anaerobic metabolisms. Our results support that the ecological success of *Pseudomonadota* is linked to the acquisition of the largest known repertoire of quinones, which allowed adaptation to oxic niches as O_2_ levels increased on Earth, and subsequent diversification into anoxic or O_2_-fluctuating environments.

## Introduction

Pseudomonadota is one of the most abundant and diverse bacterial phyla on Earth. It is composed of a large part of the former *Proteobacteria* phylum, namely the *Alpha*-, *Beta*-, *Gamma*-, and *Zeta*- *proteobacteria*, *Magnetococcia*, *Hydrogenophilia*, and *Acidithiobacillia* [1–5]. *Pseudomonadota* species are found in very different environments and dominate entire ecosystems, such as diverse aquatic environments and various types of soils or microbial mats [6–9]. Some species are involved in biotic associations, colonizing host-associated niches in the context of e.g. the rhizosphere and phyllosphere, the gut microbiota, or even in closer interactions such as endosymbiosis and intracellular lifestyle [10–13]. *Pseudomonadota* also contains genera of agricultural or medical importance such as *Agrobacterium*, *Acinetobacter*, *Pseudomonas* or *Escherichia* to name a few, making it one of the most-studied phyla. This diversity of niches is reflected by the diversity of metabolisms found in Pseudomonadota, with some lineages containing autotrophic or heterotrophic phototrophs and bacteria capable of aerobic and anaerobic respiration. The phylum also includes denitrifiers and methylotrophs, and contributes significantly to several elemental biogeochemical cycles including carbon, nitrogen, sulfur, manganese, arsenic, and iron [14–19].

The ability of bacteria to colonize diverse ecological niches is closely linked to their capacity to use various substrates for growth. This requires a large arsenal of bioenergetic enzymes, particularly dehydrogenases and reductases, which are the entry and exit points for electrons in the electron transport chains (ETC) that sustain ATP production through oxidative phosphorylation. The study of dehydrogenases and reductases has provided insights into the diversification of energy metabolism [20]. However, most of these bioenergetic enzymes form extended superfamilies, such as dimethyl sulfoxide reductases or heme-copper oxidoreductases, which complicates the elucidation of their evolutionary history and the distinction between closely related homologs with different functions [21–26].

Isoprenoid quinones transfer electrons between dehydrogenases and reductases, and are therefore central components of the ETC. The quinone types and redox potentials are adapted to the electron donors and acceptors used in the ETC, linking the repertoire of quinones to the various energy metabolisms. Nevertheless, the evolutionary dynamics of quinones repertoire has not been investigated in detail. Quinones are commonly classified into low potential quinones with redox midpoint potentials of ~ − 80 mV, such as that of menaquinone (MK), and high potential quinones with redox midpoint potentials of ~ + 100 mV [27]. High potential quinones include plastoquinone (+80 mV) found specifically in oxygenic photosynthetic organisms, sulfolobusquinone and caldariellaquinone (+106 mV) found exclusively in archaea, and ubiquinone (UQ, +110 mV) found exclusively in *Pseudomonadota* and *Eukaryota* [27–29]. MK is the most widespread quinone present in bacteria and archaea, and is thought to be ancient and generally associated with anaerobic processes [30, 31]. In contrast, high potential quinones are thought to have arisen in response to the Great Oxidation Event (GOE), and are generally associated with aerobic processes [27, 31]. The GOE, which occurred ~2.4 billion years ago as a consequence of oxygenic photosynthesis by Cyanobacteria, led to the expansion of aerobic metabolism and caused a bioenergetic revolution [32]. Indeed, the use of O_2_ as a terminal electron acceptor allowed the ETC to generate more ATP than with other previously available electron acceptors. O_2_ also had a negative impact by oxidizing previously available electron donors and the low potential quinone MK, leading to electron leakage from the ancient ETC and the production of reactive oxygen species [27]. This created a strong selective pressure for the evolution of high potential quinones that are relatively insensitive to oxygen, and for the adjustment of the redox potentials of the entire ETC to make use of the redox span between electron donors and O_2_ [27].

The phylum *Pseudomonadota* is mostly composed of aerobes and had great evolutionary success in oxic environments [33, 34]. Pseudomonadota have the widest variety of quinones known to date, with the high potential UQ, the low potential quinones MK and rhodoquinone (RQ, −63 mV), the demethylmenaquinone (demethyl-MK) with an intermediate potential of +40 mV [30], and even a methylated form of MK (methylmenaquinone, −150 mV) found only in a few members of *Shewanellaceae*, *Ferrimonadaceae*, or *Sutterellaceae* [35]. However, the types of quinones synthesized by individual species have only been characterized for some cultivated species [33, 34, 36], such as *E. coli*, which produces UQ, MK and demethyl-MK, and regulates the synthesis of these quinones in response to O_2_ levels [37, 38]. Overall, we currently lack a global understanding of the quinone repertoire across *Pseudomonadota* and of its potential role in the ecological success of this phylum.

Recent contributions, including our own [39–41] to the identification of enzymes contributing to quinone biosynthetic pathways now allow the inference of the genetic potential for quinone biosynthesis in a given species. In this study, we have conducted a comprehensive survey of the quinone pathways in Pseudomonadota from genomic and phylogenetic analyses. The findings lead to a detailed evolutionary scenario of the quinone repertoire of *Pseudomonadota* in the context of their metabolic diversification and of Earth oxygenation.

## Materials and methods

### Genome dataset

We downloaded 19 777 complete genomes of the phylum Pseudomonadota available at the NCBI (National Center for Biotechnology Information) database, last accessed in August 2023 [42]. Taxonomic assignments were retrieved from the NCBI taxonomy and the Genome Taxonomy Database (release 220) [43–45]. A selection of one genome per species (from NCBI species ID) was performed using the following criteria by decreasing order of priority: (i) NCBI “reference” genome, (ii) NCBI “representative” genome, (iii) at random. After selection we obtained 4107 *Pseudomonadota* genomes (Table S1). A dataset representative of the prokaryotic diversity was selected and downloaded using the same procedure, resulting in an additional set of 5392 non-*Pseudomonadota* complete genomes. We also downloaded from the NCBI all available genomes of *Magnetococcales* (NCBI order, last accessed November 2023) and all genomes of *Zetaproteobacteria* (NCBI class, last accessed February 2024). We kept high-quality genomes under the “MIMAG” criteria (completeness above 90% and contamination under 5%) [46]. We counted a total of 33 *Magnetococcales* and 26 *Zetaproteobacteria*. Coding sequence annotation was performed for all selected genomes with Bakta (v1.9.3) using the full database [47].

### Annotation of quinone proteins in the dataset

To identify proteins from the MK (Men and futalosine) pathway, O_2_-dependent and O_2_-independent UQ pathways, and the RQ biosynthetic pathway, hidden Markov model (HMM) profiles were used (Fig. S1). We employed previously published HMM profiles for UbiA, MenA, UbiE (MenG), UbiT, UbiU, UbiV, U32 proteases, Coq7, UbiF, UbiH, UbiI, UbiL, UbiN, UbiM, and flavin monooxygenases [40, 41, 48]. In addition, we designed HMM protein profiles for the remaining UQ enzymes, the two MK pathways, and the plastoquinone pathway, using the same approach as before (see Methods S2 for details).

Finally, the HMM protein profiles were used to annotate our dataset of *Pseudomonadota*, additional *Magnetococcales*, *Zetaproteobacteria*, and prokaryotic genomes with *hmmscan* (HMMER suite v3.3.2) [49]. The following parameters were used for hits selection: hits above GA scores, or i-evalue below 0.001 and profile coverage above 0.5 (alignment length over sequence length) when GA scores were not defined in the profile. In cases where multiple profiles matched a sequence, the hit with the lowest i-evalue was selected for annotation. All profiles used in this study are available as Supplementary Dataset S1, as well as all genome annotations (Tables S1, S5).

### Phylogenetic analyses of pathways

For phylogenetic analysis of the pathways of interest we used four datasets. (I) We set up a “base” dataset of genomes covering the diversity of *Pseudomonadota*, considering the presence of the quinone pathways of interest, the sequence diversity, and the taxonomy. To account for the diversity, we first selected sequences of UbiB, this gene being the most conserved in *Pseudomonadota*. We clustered the UbiB sequences using MMseqs2 [50] (parameters: 70% sequence identity and 80% coverage) and picked one UbiB sequence from each of the clusters, resulting in 346 sequences. To have a balance between taxonomic classes, we randomly selected some representatives from a sub-sample of taxonomic orders in each class. To follow the Men pathway evolution, we expanded the above selection to include seven *Magnetococcia* and *Gammaproteobacteria* genomes lacking UbiB but having the Men pathway. This resulted in a total of 107 selected genomes. This base dataset was used for the phylogenetic analysis of the following UQ proteins: UbiA, -C, -D, −G, -U, and -V. (II) For UbiE, −T/J, and -B phylogenies, the dataset was enriched with additional *Zetaproteobacteria* sequences. (III) For Men phylogenies—MenB, MenD and MenF—we expanded the *Pseudomonadota* base dataset by adding sequences of Men proteins from up to three randomly selected genomes per prokaryotic class (NCBI taxonomy classes). (IV) We also made a RquA phylogenetic tree using every hit obtained in the original dataset.

From each genome of the datasets, we collected the annotated quinone protein sequences. Except for the RquA phylogeny, when several hits were present for a given protein, a single sequence was selected based on its proximity to other genes from the same pathway. Each phylogenetic tree was constructed from a multiple sequence alignment (MSA) produced with MAFFT using the l-ins-i algorithm (v7.487) [51]. Sites filtering of the MSA was performed using BMGE (v 2.00) using the default parameters and the BLOSUM30 matrix [52] except for RquA, where it was done with Clipkit (v 1.4.1, “kpic” algorithm) [53]. The maximum likelihood trees were obtained with IQ-TREE [54] (v 2.2.0.3) using the best evolutionary model selected by ModelFinder (option “-MFP”, BIC criterion used) [55]. Both ultrafast bootstraps (UFBoot) and SH-aLRT branch supports were computed using 1000 iterations [56, 57]. We ran several iterations of tree reconstructions with IQ-TREE, the first one allowing to select the best model. This model was reused in 10 extra iterations, among which we chose the one that showed the most frequently found topology and the highest support in deep nodes. When necessary, sequences provoking long branches that could perturb tree reconstructions were removed from the analyses and phylogenetic analyses reiterated. For each pathway, rooted phylogenies were built using corresponding outgroups.

### Species tree

We constructed a species tree for 107 *Pseudomonadota* genomes selected for the gene phylogenies and six genomes from *Myxococcota* and *Bdellovibrionota* to serve as outgroups. This was done by concatenating 41 marker proteins identified using MarkerFinder [58]. One genome containing less than 90% of markers was discarded, resulting in a total of 112 genomes. We used MAFFT (l-ins-i algorithm) [51] to produce MSA with the obtained sequences for each marker individually and selected informative sites with Clipkit (“kpic” algorithm) [53]. The MSA concatenation and tree inference was performed by IQ-TREE (v 2.2.0.3) with the options “-p” for the concatenating of all the filtered alignments and “-B 1000” for the computation of bootstrap supports [54, 56, 59], and the best model was computed for each marker protein individually using ModelFinder [55]. We constructed the *Gammaproteobacteria* species tree presented in supplementary data (Fig. S2) using the same procedure.

### Phylogenetic reconciliations

To infer evolutionary events that occurred along the evolution of the proteins from the Men pathway, individual protein trees were built as presented above. Each of these were reconciled with a species tree enriched with Prokaryotic species using the GeneRax software [60] with the option “UndatedDTL” that accounts for duplications, transfers, and losses. In each case, the input MSA was provided, and the reconstructed protein tree was used as a starting tree for the reconciliation process. Reconciliation scenarios were then analysed after visualisation with the Thirdkind software [61]. Reported events correspond to those that were inferred by the majority of the individual reconciliations.

### Gene context analyses

We extracted the genetic positions of genes of interest from the “GFF” files obtained from Bakta genome annotations [47]. We constructed adjacency matrices for each pathway (Figs. 4B, S3), in which two genes were considered as contiguous if they were positioned less than 500 bp apart. We plotted the corresponding graphs with Cytoscape [62]. The thickness of the lines connecting the gene nodes depicts a contiguity score that is calculated as in [63]: for two given genes, the score corresponds to the number of times they are found to be contiguous, divided by the number of occurrences of the rarest of them.

For the quantification of the conservation of proximity between any two genes, we follow up on a previous study [64] and define, for each pair of genes $\left(i,j\right)$, a degree of synteny as a relative entropy:


$$ {D}_{ij}={f}_{ij}\mathit{\ln}\frac{f_{ij}}{p}+\left(1-{f}_{ij}\right)\mathit{\ln}\frac{1-{f}_{ij}}{1-p} $$

In this equation, ${f}_{ij}$ stands for the number of genomes two genes are found in proximity along the DNA. To this end, for a given chromosome of size $L$, the DNA proximity is set by a distance $d=p\times L/2$ such that the probability of finding two genes at a distance lower than $d$ in the case of a random positioning of the genes along the chromosome is equal to $p$ (that of the equation). We choose $p=0.02$ as in [64]; thus, we have $d=50 kb$ and $d=5 kb$ respectively for chromosomes of size $L=5 Mb$ and $L=500 kb$. In this context, pairs of genes that are not in a synteny relationship are expected to have a value of ${f}_{ij}$ close to $p$ and, hence, a value of ${D}_{ij}$ close to $0$. In contrast, genes in a synteny relationship are expected to have a value of ${f}_{ij}$ that is much larger than $p$, leading to a large value of ${D}_{ij}$ (Fig. S4).

To analyze the co-occurrence tendency of two genes within the same pathway across genomes, we constructed a covariance matrix (Fig. S5).

### Motif discovery and scanning for FNR binding sites

A motif discovery search was launched 450 bp upstream from the start codon of each of the annotated genes *ubiT, -U, -V* using the program MEME [65, 66] (MEME suite v 5.3.3). As previously proposed for FNR binding site search [67], the search parameters were set to identify up to five palindromic motifs of 14 bp present in 0 or 1 copy on the given strand. Resulting consensus sequences were compared to prokaryotic transcription factors databases (CollecTF, Prodoric, RegTransBase) using Tomtom [68] (MEME suite). To account for a potential motif variability across Pseudomonadota, we performed motif discovery and collected the resulting consensus motifs for each major class separately (*Alpha*-, *Beta*-, and *Gamma*- *proteobacteria*) in addition to the consensus motif obtained for the full dataset. Eventually, all 450 bp sequences of the dataset were scanned with the consensus motifs retrieved using the scanning program FIMO [69] (MEME suite) to retrieve all the possible matches on the sequences with the following parameters: *P* value <1e-5, q-value ≤0.05.

### Growth of *Magnetococcus marinus* strain MC-1

Cells were grown microaerobically in a semi solid oxygen-gradient medium, in autotrophic conditions with thiosulfate used as electron donor and oxygen as electron acceptor. The medium was prepared according to [70]. Aerotactic bands of cells were harvested from exponential and stationary phase cultures. Cells were immobilized by adding 25 μM of a solution of the flagellar motor inhibitor carbonylcyanide m-chlorophenyl hydrazon, counted in a Mallasez counting chamber and stored at −20°C before quinone analyses.

### Measurements of isoprenoid quinones in *Magnetococcus marinus* strain MC-1

Suspensions of 1^*^10^8^ cells in 300 μl growth medium were transferred to glass tubes by pipetting 3^*^1 ml methanol. We then added 10 μl 3 M KCl, 2 μl 4 μM MK_7_ (as an internal standard), and a volume of 200 μl glass beads (0.5 mm), and vortexed for 1 min. 2 ml petroleum ether (boiling range 40–60°C) was added and the sample was vortexed for 1 min. The upper phase was transferred to a fresh tube and the methanol phase was extracted again with 2 ml petroleum ether. The two petroleum ether phases were combined and evaporated under a flow of nitrogen. The dried extracts could be stored at −20°C until High Performance Liquid Chromatography (HPLC) analysis. The dried extracts were suspended in 100 μl ethanol and a volume corresponding to an initial quantity of 5^*^10^7^ cells was injected onto the HPLC. HPLC-electrochemical detection-mass spectrometry was performed as described previously [41]. Standard curves were generated in the same conditions by injecting various volumes of a solution containing 6 μM UQ_8_, 8 μM MK_7_, and 12 μM UQ_10_ (all from Sigma).

### O_2_ reductases annotation

To identify the presence of heme-copper oxygen reductases or cytochrome *bd* oxygen, we used the HMM profiles published in [21, 71]. The cyt *bd* profiles used were OR-C, OR-N, qOR1, qOR2, qOR3 and qOR4a whereas the heme-copper oxygen reductases profiles used were A, B1–9, BNOR, C, CNOR, coxBCuA, coxBqox, ENOR, GNOR, NNOR, NOD, QNOR, SNOR. When a profile was available for a subunit II, we also used it. The search was carried out by *hmmscan* (HMMER v3.3.2) [49] with an i-evalue threshold of 1e-5 and a sequence coverage threshold of 0.5.

### Metabolism annotation

We used a table generated in [72], which gathers phenotypic traits of bacterial and archaeal species from different sources. We extracted information on organism metabolism when species names matched between the dataset of 4107 genomes of *Pseudomonadota* and this table. Of the 1401 species in common between the two datasets, metabolic information was provided for 1084 of them. Compared to the original table, we grouped together species annotated as “obligate aerobic” and “aerobic” into an “aerobic” category, as well as “obligate anaerobic” and “anaerobic” into an “anaerobic” category. *Magnetococcus marinus* was reassigned from aerobe to microaerophile [70].

### Statistical analyses

The significance of the association of the presence of the O_2_-independent UQ pathway and of the Men pathway was assessed in a phylogenetic framework by comparing the likelihood of the “Discrete” and “DiscreteIndependent” models from BayesTraits (v 4.1.2) with a Likelihood Ratio Test [73]. The likelihoods of the two models were estimated by maximum likelihood. This test was performed on a subset of the genomic dataset (179 species) that was randomly sampled, and for which a rooted species tree was built as described above.

The presence of a phylogenetic signal for the metabolism data was evaluated on the same subset of 179 genomes using the Delta statistic that is dedicated to categorical data [74, 75]. In this procedure, ancestral state characters were estimated using PastML [76]. The Delta statistic was then computed for the metabolism data using the 179 species tree and a null distribution of the statistic was computed based on 10 000 randomizations of the metabolism data within the dataset, allowing the computation of a *P* value.

A permutation test was designed to test the significance of the association between the two categorical traits: presence of quinone pathways and type of O_2_ metabolism, across the entire set of annotated genomes (see Methods S3, [77]).

## Results

### Distribution of quinone pathways in *Pseudomonadota*

Two distinct pathways (termed futalosine and Men) have been identified for the production of MK (Fig. S1, Text S1). UQ biosynthesis occurs via two partially overlapping pathways (Fig. S1, Text S1), an O_2_-dependent pathway and an O_2_-independent pathway that we discovered recently [41, 42]. RQ synthesis proceeds from UQ in a single step catalysed by the RquA protein [43], which we confirmed experimentally in 15 species (Figs. S6, S7, Text S2, Methods S1, Table S2).

**Figure 1:**
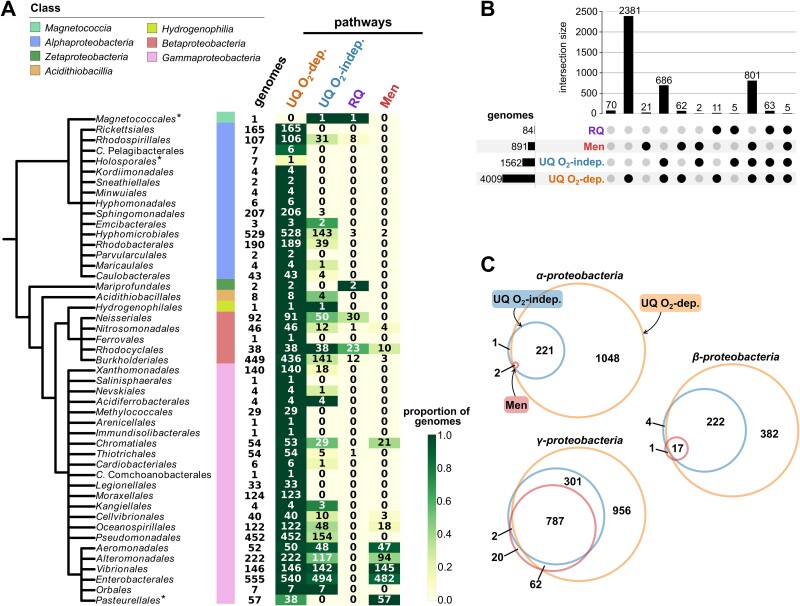
**Quinone pathways distribution and co-presence in *Pseudomonadota*.** (**A**) Quinone pathways distribution in orders of *Pseudomonadota*. Along the schematic species tree of the *Pseudomonadota* are shown the corresponding classes and the number of complete genomes for which the order information in the NCBI taxonomy is specified. Next, the number of genomes with UQ, RQ, and men (MK production) pathways is specified on a green gradient, indicating the proportion of genomes with each pathway in the orders. Orders marked by an asterisk (^*^) are the ones displaying a lesser presence of the UQ O_2_-dependent pathway as compared to the overall distribution. (**B**) Co-presence of the UQ, RQ, and men (MK production) pathways in *Pseudomonadota*. Dots filled in black indicate the presence of a pathway and the number of genomes with each pathway is displayed on the bottom left. The number of times pathway combinations are found in the genomes is shown above the corresponding bars. (**C**) Joint presence of the men pathway for MK production, UQ O_2_-dependent and UQ O_2_-independent pathways in *Alpha*-, *Beta*-, and *Gamma*- *proteobacteria*.

To determine the occurrence of these quinones in Pseudomonadota, we used previously designed HMM profiles and constructed new profiles to have all enzymes involved in the corresponding biosynthetic pathways represented. Obtaining highly reliable profiles involved the combined usage of phylogenetics, gene synteny and experimental validations (this study and [40, 41]). We then established thresholds for the number of enzymes required to infer the presence of the pathways from genomes. This was based on the bimodality of the distribution of the number of biosynthetic genes in genomes (Fig. S8, Text S3, Methods S2). In total, we annotated the MK, UQ and RQ biosynthetic pathways in a dataset consisting of the complete genomes from 4107 *Pseudomonadota* species and 5392 non-*Pseudomonadota* prokaryotic species. The UQ O_2_-dependent pathway is present in almost all species of *Pseudomonadota* (~98% of the dataset) and appears to be absent outside this phylum (Fig. 1A, Fig. S8, Table S1). This observation is consistent with the hypothesis that the ability to produce UQ aerobically originated in the common ancestor of the *Pseudomonadota* [40]. The UQ O_2_-independent pathway is distributed throughout the *Pseudomonadota* species tree (Fig. 1) but exhibits less conservation than the O_2_-dependent pathway. It is only present in 39% of the genomes from our dataset (17% of the *Alphaproteobacteria*, 38% of the *Betaproteobacteria,* and 50% of the *Gammaproteobacteria*). In accordance with a previous study [78], the RQ pathway is sparsely distributed, being present only in 84 genomes across Pseudomonadota. It is predominantly found in *Alpha*- and *Beta*- proteobacteria, with only one *Gammaproteobacteria* species (*Candidatus Thiothrix singaporensis*) displaying the RQ pathway.

We did not find the futalosine pathway in *Pseudomonadota* and therefore conclude that demethylmenaquinone or MK can only be produced via the Men pathway in this phylum (Texts S1 and S3). The Men pathway was predominantly identified in *Gammaproteobacteria*, specifically in *Aeromonadales*, *Alteromonadales*, *Vibrionales*, *Enterobacterales,* and *Pasteurellales*. Of the 871 occurrences of the Men pathway, 825 were observed within these orders. Overall, 20% of the *Pseudomonadota* species demonstrated potential for demethylmenaquinone or MK production.

### UQ O_2_-independent pathway shows a strong co-distribution with the low potential quinone pathways

We investigated the quinone pathways co-distribution. Given the high prevalence of the UQ O_2_-dependent pathway, it globally co-occurs with any other quinone pathways that are present in genomes. In several cases, whenever the UQ O_2_-dependent pathway was absent, another quinone pathway was found: the O_2_-independent pathway in the *Magnetococcales* and the Men pathway in *Pasteurellales* (Fig. 1A). There were nevertheless 70 species without any detected quinone pathways (Fig. 1B), most of which are ectosymbionts or endosymbionts with reduced genomes (Table S1). The *Holosporales*, a clade fully represented by protists’ symbionts [33], display an incomplete UQ pathway for 6/7 species, which is consistent with previous observations [79]. Whenever the UQ O_2_-independent pathway was found without the O_2_-dependent pathway, a low potential quinone pathway was always present (Fig. 1B). Strikingly, the Men pathway was frequently found in association with the UQ O_2_-independent pathway (808 species), whereas it was rarely found solely with the UQ O_2_-dependent pathway (62 species, Fig. 1B-C). This association was significant when accounting for phylogeny (Likelihood Ratio Test, *P* value <10e^−10^). 21 species possess the Men pathway as their unique quinone pathway (Fig. 1B-C), almost all of them belonging to the *Pasteurellales* (Fig. 1A). This clade comprises mostly opportunistic pathogens known to be facultative aerobes [34]. Most of these Pasteurellales lack UbiE and must therefore produce demethyl-MK—a quinone with a higher redox potential than MK (Table S1, Fig. S1). This is consistent with demethyl-MK production by species of the genera *Haemophilus* and *Aggregibacter* as reported in the literature [80,81]. RQ frequently occurs with the UQ O_2_-independent pathway (73 cases for 84 genomes annotated with RquA) and is seldom associated with MK (5 cases) (Fig. 1B). This reflects the fact that the two low potential quinones RQ and MK are rarely found together in the same species. Overall, we found that the newly discovered UQ O_2_-independent pathway is usually associated with the O_2_-dependent UQ pathway and is significantly enriched in genomes harbouring low potential quinones (876 genomes have both a low potential quinone and UQ O_2_-independent pathways, representing ~90% of the genomes with low potential quinones). This suggests distinct functions for UQ and low potential quinones under O_2_-deprived conditions, in agreement with our recent finding of the contribution of the UQ O_2_-independent pathway to the adaptation of *E. coli* to changing respiratory conditions [82]. It was therefore of interest to evaluate the structure and regulation of the UQ O_2_-independent pathway across *Pseudomonadota*.

### Compact *ubiTUV* genetic loci and FNR binding sites suggest a conserved FNR regulation of the UQ O_2_-independent pathway

**Figure 2:**
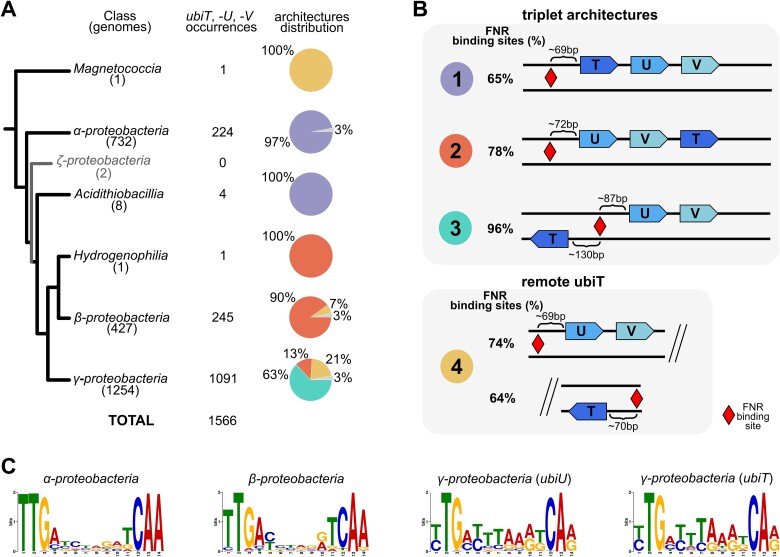
**Genetic architecture of *ubiTUV* and conservation of an FNR binding site across *Pseudomonadota*.** (**A**) Distribution of *ubiTUV* genetic architecture across *Pseudomonadota*. For each class of *Pseudomonadota* are depicted the number of genomes analysed, the number of *ubiTUV* occurrences, and the proportion of each genetic architecture (represented by pie charts), as described in panel B. Architectures with a low abundance (under 3% of the total *ubiTUV* occurrences of the class) are depicted in gray in the pie charts. (**B**) Main genetic architectures and position of the candidate FNR binding sites. In architectures “1”, “2”, and “3”, *ubiTUV* are immediately next to one another (< 500 bp apart) whereas *ubiT* is distant from *ubiUV* in architecture “4”. FNR binding sites are represented by red diamonds and their median distance from the start codon of the nearest gene is specified. The percentage of candidate FNR binding sites detected in each architecture is indicated before the representation of the three genes. (**C**) MEME logo corresponding to the motifs identified as FNR binding sites in each class.

The O_2_-independent pathway is defined by the presence of the genes *ubiT*, *-U,* and *-V* (*ubiTUV)* and is found in 1562 genomes, four of which contain two copies of the three genes, giving a total of 1566 occurrences of the *ubiTUV* genes (Fig. 2A). Our analyses show that the three genes predominantly co-localize (in ~83% of cases) and that *ubiU* and *ubiV* are less than 500 bp apart in 99.6% of the cases (distance greater than 500 bp for only seven cases, Table S3). The co-localization of *ubiT*, *-U,* and *-V* is significantly higher than reported in 2019 [41] as the genes analysed then were not all ordered along the chromosome, which resulted in an underestimation of the genes co-localization. The genetic organization of *ubiTUV* is highly conserved as *ubiV* always follows *ubiU; ubiT* is located either upstream or downstream of the conserved *ubiUV* pair on the same strand or on the complementary strand (Fig. 2B, Table S3). Therefore, four main genetic architectures of *ubiTUV* (out of 18 possible) are found across *Pseudomonadota* and their distribution follows the species tree (Fig. 2A, Text S5). The strong conservation of the *ubiTUV* genes at a single locus suggests a co-regulation process. Therefore, we investigated the sequences upstream of the *ubiTUV* genes.

We used the MEME suite [65, 66, 68, 69] to search and identify potential regulatory motifs in the 450 bp sequences upstream of the *ubiTUV* genes in a two-step process (untargeted first, then specifically, see Materials and Methods). We identified FNR (the “fumarate and nitrate reductase” global anaerobic regulator) binding sites as the most significant motifs in the sequences upstream of *ubiU* and *ubiT*. FNR is an O_2_-sensing master regulator of anaerobiosis, which activates the transcription of *ubiTUV* in *E. coli* [82]. Eventually we found at least one candidate FNR-binding motif upstream of *ubiU*, *ubiT,* or both, for 1362 out of 1566 *ubiTUV* triplets (87%). 1001/1566 *ubiT* and 1180/1566 *ubiU* genes had at least one FNR site predicted upstream of their sequence, whereas *ubiV* had none (Table S4). Whenever *ubiTUV* are on the same strand (architectures “1” and “2”), FNR-binding sites are predicted mostly before the first gene of the triplet with frequencies of 78% and 65% for respectively *ubiU* and *ubiT* (Fig. 2B). With architecture “3” where *ubiT* and *ubiUV* are on opposite strands and directions, 96% of the cases show a predicted FNR site between *ubiU* and *ubiT*. Regarding architecture “4” where *ubiT* is found apart from *ubiUV*, the candidate sites tend to be located before *ubiU* in 74% of the cases and/or *ubiT* in 64% of the cases (Fig. 2B). The median position of the sites in all lineages could be indicative of a positive control (Fig. 2B, Fig. S9). In *Gammaproteobacteria* in particular, the position of the candidate sites relative to the start codon was consistent with that found in *E. coli* when FNR acts as a positive regulator [83] (Fig. 2B, Fig. S9, Text S5). The role of FNR or FNR homologs in the expression of the UQ O_2_-independent pathway has been formally demonstrated only in *E. coli* [82] and *P. aeruginosa* [84], and is highly suggested from expression data in the alphaproteobacterium *Sphingopyxis granuli* [67]. Overall, our results support a conserved role of FNR in the joint positive regulation of *ubiT, -U, -V* throughout *Pseudomonadota*, resulting in the activation of the UQ O_2_-independent pathway when O_2_ levels decrease.

### Magnetococcia rely on the O_2_-independent pathway for UQ (and RQ) production

The candidate class *Magnetococcia* is presented in most phylogenies as a sister lineage of *Alphaproteobacteria*, or as a deeply-branching lineage within *Pseudomonadota* [85–90]. The quinone pathway combination of *Magnetococcia* stands out from *Alphaproteobacteria* because *M. marinus*, the only representative in our dataset, lacks the UQ O_2_-dependent pathway but harbours the O_2_-independent one (Fig. 1A). To verify whether this pattern is specific to *M. marinus* or is representative of the entire class, we expanded our genomic dataset to include 32 high quality *Magnetococcia* genomes that were not fully assembled, most of them being metagenomes assembled genomes (MAGs) with a very high level of completeness [46] (Table S5). The analysis of these genomes showed that all of them lack the UQ O_2_-dependent pathway whereas 28 genomes possess the O_2_-independent pathway and 30 display the *rquA* gene (Table S5). We showed that the RquA protein from *M. marinus* was functional in *E. coli* (Fig. S6), which questioned a previous report that UQ_10_ is the only isoprenoid quinone in *M. marinus* [70]. We therefore grew *M. marinus* and established that its quinone profile in exponential and stationary growth phases showed two distinct peaks corresponding to RQ_8_ at 6.8 min and to UQ_8_ at 7.8 min, whereas UQ_10_ was undetectable (Fig. S10A-B). The identity of UQ_8_ and RQ_8_ was confirmed by mass spectroscopy (MS) analysis (Fig. S10C-F) and weak signals corresponding potentially to RQ_7/9_ and UQ_7/9_ isoprenologs were also detected (Fig. S10G-J). Overall, our results establish that *M. marinus* synthesizes predominantly RQ_8_ and UQ_8_, and not UQ_10_ as previously reported. This is consistent with the presence of the UQ and RQ pathways inferred from most of the *Magnetococcia* genomes included in our analysis. In addition to UQ and RQ, we found the Men pathway in one MAG and the futalosine pathway in another MAG, although only five genes were detected. A very few other genomes seem to have conserved a small number of genes from the futalosine pathway (Table S5). Inspection of the contigs containing the Men and futalosine genes showed the presence of genes conserved throughout *Magnetococcia*, suggesting that these MK genes are not contaminants.

All *Magnetococcia*’s genomes with *ubiTUV* displayed the architecture “4” (Figs. 2B, S11, S3), but we could not find significant FNR binding sites upstream any of the three genes. We further noticed that *ubiTUV* intertwined with other genes involved in UQ biosynthesis (Fig. S11, Table S5) as opposed to other *Pseudomonadota* (Fig. S3). Overall, we conclude that magnetococcia have the genetic potential to produce UQ and RQ through a constitutively expressed UQ O_2_-independent pathway, as experimentally demonstrated for *M. marinus*.

### Both UQ biosynthetic pathways were vertically inherited from the last common ancestor of *Pseudomonadota*

**Figure 3:**
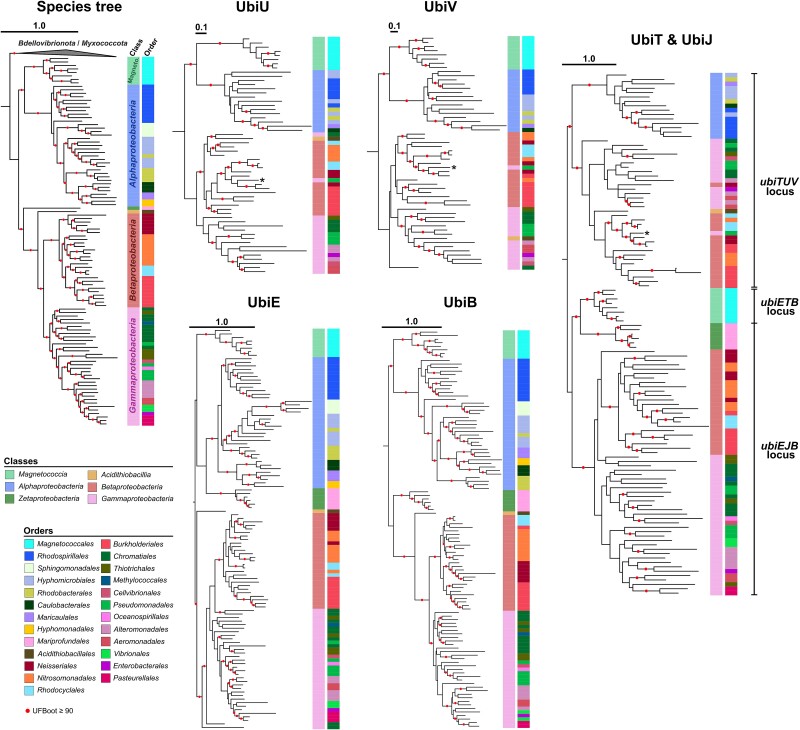
**Phylogenies of the UbiU, UbiV, UbiE, UbiB, UbiT, and UbiJ proteins in *Pseudomonadota*.** The species tree made from the concatenation of 41 marker genes of a subsampling of *Pseudomonadota* was rooted using an outgroup of *Bdellovibrionota* and *Myxococcota* sequences. The branches with high support (UFBoot ≥95%) are indicated by red dots. The other trees are phylogenies of the UQ genes that are the most conserved at a single locus: *ubiTUV* and *ubiEJB* (*ubiETB* for *Magnetococcia*). The sequences come from the selected species’ genomes. UbiU, -V, −E, and -B trees are rooted as to follow the species tree, and rooted phylogenies of Ubi proteins presented in Fig. S14. UbiT and UbiJ are presented on a same phylogeny. On this tree, the root is placed to separate the sequences from the *ubiTUV* locus from the sequences of the *ubiEJB* locus (*ubiETB* for *Magnetococcia*). Every tree displays the class- and order-level taxonomy. In UbiE, -B, and -T & -J trees, more sequences from *Zetaproteobacteria* have been added. *Entomomonas moraniae* which acquired *ubiTUV* via an HGT event is indicated by a “^*^” on the respective trees. The trees of UbiU, UbiV, UbiE, UbiB, and UbiT & -J were obtained from the analysis of 312 (230), 260 (225), 236 (190), 413 (372), and 92 (90) aligned positions (informative sites) respectively using IQ-TREE with Q.Pfam+R5 as the best selected model for UbiU, LG + F + I + I + R5 for UbiV, Q.Pfam+R7 for UbiE, LG + F + I + I + R6 for UbiB, Q.Pfam+F + G4 for UbiT & UbiJ. Tree scale bars are expressed in number of substitutions per site. The branches with high support (UFBoot ≥90%) are indicated by red dots.

The dissemination of the UQ O_2_-independent pathway across the entire diversity of the *Pseudomonadota* raises questions about the origins and evolution of the *ubiTUV* genes. Contrary to other UQ genes, both *ubiTUV* and *ubiEJB* showed a strong pattern of conservation at a single locus (Figs. S3, S4). *ubiJ* is missing in *Alphaproteobacteria* and *Magnetococcia* (Tables S2, S5, Figs. S12, Text S4), and in *Magnetococcia* the *ubiT* gene is flanked by *ubiB* and *ubiE*, which is the position of *ubiJ* in *Beta*- and *Gamma*- proteobacteria. UbiJ plays an important role in the stability of the aerobic UQ biosynthesis complex, which consists of seven proteins (UbiE-K) in *E. coli* [91]. The function of the UbiT protein is not fully understood, but UbiT and UbiJ are homologs and share the SCP-2 (Sterol Carrier Protein-2) domain, which makes up most of the sequence of these rather small proteins (~180–200 amino-acids). In order to increase the phylogenetic resolution of our analyses, we enriched the dataset with a few high-quality genomes and MAGs of *Zetaproteobacteria* and built a phylogenetic tree with UbiT and UbiJ. The phylogeny of UbiT and UbiJ can be split in two subtrees that separate completely the sequences from the loci *ubiTUV* and *ubiEJB* (*ubiETB* for *Magnetococcia*) and reflect the overall species tree of the *Pseudomonadota*. We thus propose that the two loci were present in the last common ancestor of the *Pseudomonadota* and were inherited vertically (Fig. 3). Although the *ubiUV* and *ubiT* genes from the *ubiTUV* locus are generally transmitted vertically, we captured an event of horizontal gene transfer (HGT) in *Entomomonas moraniae* that acquired *ubiT*, *-U* and *-V* from a member of the *Neisseriaceae* (*Betaproteobacteria*) (Fig. 3). The *ubiU*, *-V*, *−E,* and *-B* gene trees follow the species tree and are congruent with each other, supporting their vertical inheritance from a common ancestor of the *Pseudomonadota* (Fig. 3). We performed additional phylogenetic analyses on other genes from the core UQ pathway and each of them displayed the same pattern of vertical inheritance (Fig. S13). Based on these analyses, we conclude that the UQ O_2_-dependent pathway and the UQ O_2_-independent pathway emerged in an ancestor of contemporary Pseudomonadota, and were both present in the last common ancestor of this phylum.

### Low potential quinones were repeatedly acquired by *Pseudomonadota* through horizontal gene transfers

**Figure 4:**
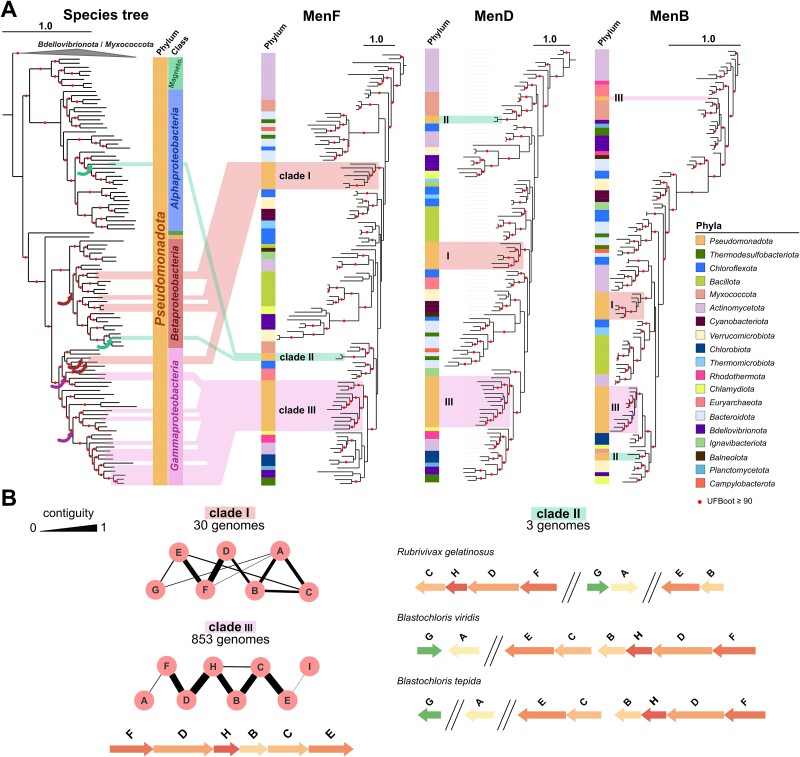
**Phylogenies of MenF, MenD, and MenB in prokaryotes.** (**A**) The species tree is the same as in Fig. 3. The other trees correspond to phylogenies of the well conserved MenF, -D, and -B proteins and display the phylum-level taxonomy. The MenF tree has been rooted with other isochorismate synthase sequences as an outgroup (see Fig. S15 for details). The other men protein trees are rooted to follow the MenF rooting (i.e. with a subgroup of *Actinomycetota*). *Pseudomonadota* species containing MK are highlighted in light purple, red, and green on the species tree. The corresponding sequences are highlighted by the same colours in the protein trees and form three separate clades (I-III). Proposed HGT events are indicated by coloured arrows on the species tree, details of the reconciliation scenario proposed by GeneRax [60] is presented in Fig. S16. The trees of MenF, MenD, and MenB were obtained from the analysis of 227, 333, and 398 aligned positions respectively using IQ-TREE. The best selected model was respectively LG + F + R7, LG + F + I + I + R6, and LG + I + I + R4. Tree scale bars are expressed in number of substitutions per site. The branches with high support (UFBoot ≥90%) are indicated by red dots. (**B**) Genetic organization in each of the three clades with letters identifying *men* genes. On the graphs, the thickness of the connecting lines corresponds to the contiguity level, as in [63]. Arrows type sketches representative of clades II and III were drawn using the GenomeViz Python library (v0.4.4) in a home-made script [93].

The Men pathway is ancient and known to be present in many prokaryotic lineages [92], but its sparse distribution in *Pseudomonadota* suggests that it was either ancestral and lost repeatedly or acquired by HGT. To differentiate between these two hypotheses, we examined the phylogeny of the Men proteins. We selected the genes *menF*, *menD,* and *menB* based on their prevalence in genomes annotated with the Men pathway and based on their genomic proximity to one another and to other *men* genes (Fig. 4). MenF, -D, and -B phylogenies were reconstructed using a selection of sequences from *Pseudomonadota* and species sampled through the tree of prokaryotes and their phylogenies were reconciled with the species tree using GeneRax [61] (Fig. S16). The phylogenies of MenF and MenD are very similar in the sense that often, sequences from the same phylum are found in sub-groups scattered across the tree (Fig. 4A). For instance, sequences of the *Chloroflexota* group in five distinct sub-clades in our trees. This is in line with the previous proposition that the Men pathway tends to spread via HGT [92]. The Pseudomonadota are no exception because their MenF, MenD, and MenB sequences group into at least three separate clades in the three gene trees (clades I to III, Fig. 4). Although the support for deep branches of these trees is generally weak, the identified clades of *Pseudomonadota* are well-supported. Furthermore, the *men* genes present different genetic architectures that are well conserved within each of the three clades (Fig. 4B). Together, these results suggest several transfer events of the Men pathway into *Pseudomonadota* that were followed in the case of ancient transfers by several differential losses. From the comparison and the reconciliation of the gene trees and the species tree, at least seven HGT events can be proposed (Figs. 4A, S2, S16).

The distribution of *rquA* is very sparse in *Pseudomonadota* and most sequences are found in *Betaproteobacteria* (Fig. 1A). The position of sequences from *Alpha*- and *Gamma*- *proteobacteria* in the phylogeny of RquA is not consistent with the species tree of *Pseudomonadota* (Fig. S6A), suggesting HGT events. Nevertheless, *rquA* sequences from *Chromobacterium* and *Aquitalea* consistently group together, suggesting a vertical transmission in these lineages. Even if it is difficult to conclude on the evolutionary history of the *rquA* gene, as its shortness and low sequence conservation result in weakly supported phylogenies (Figs. S6A, S7), the distribution of this gene seems to result from a combination of vertical and lateral transmission events.

### Species O_2_ requirement varies according to quinone pathways repertoire

**Figure 5:**
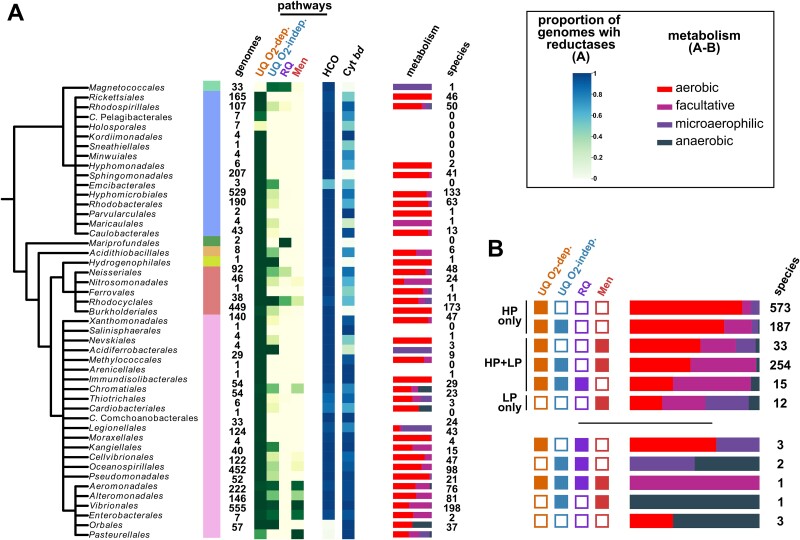
**Oxygen requirement and O**_**2**_  **reductases annotation (Heme-copper oxygen reductase and cytochrome *bd*).** (**A**) Along the schematic species tree of the *Pseudomonadota* is represented the quinone pathways distribution as shown in Fig. 1A. The reductase annotations obtained are depicted by a light-green to dark-blue gradient. Next, the type of metabolism for species annotated in the dataset from [72] is mapped (aerobic in red, facultative in magenta, microaerophilic in purple, anaerobic in dark blue). The number of species for which the information was available is indicated in the column “species”. (**B**) For each quinone pathway combination, the proportion of each metabolism (aerobic, facultative, microaerophilic, anaerobic) is shown with the same colour code as in panel A. The column “species” contains the number of species for each combination. Quinone pathways combinations found in more than 10 genomes are sorted in descending order of aerobes proportion. The other combinations are shown below.

Heme-copper oxygen reductases and cytochrome *bd* oxygen reductases (Cyt *bd*) are the two main enzymatic families used by bacteria for aerobic respiration, reducing O_2_ with electrons provided by quinones directly or indirectly [21, 71]. Cyt *bd* has a high affinity for O_2_ [94–96] and has increased expression under microoxic conditions [94,97,98]. Therefore, we hypothesized that Cyt *bd* might be associated with the UQ O_2_-independent pathway or low potential quinones. To evaluate the distribution of O_2_ reductases in Pseudomonadota, we used HMM profiles developed in recent studies [21, 71]. The vast majority of *Pseudomonadota* possess at least one heme-copper oxygen reductase (~97%), although two orders (*Orbales* and *Pasteurellales*) showed none (Fig. 5A, Table S6). Cyt *bd* was more sparsely distributed but did not show any obvious association to the presence of the quinone pathways (Fig. 5A). Therefore, we conclude that the quinone types do not influence the composition in O_2_ reductases in *Pseudomonadota*.

Low potential quinones are typically associated with anaerobic metabolism and high potential quinones with aerobic metabolism [30, 31, 37], although this has not been documented on a large scale in clades possessing both types of quinones. Therefore, we sought to annotate the respiratory metabolism of *Pseudomonadota* and investigate a possible link to the quinone pathways they possess. We collected the metabolic information from a database manually curated in 2020, which included ~25% of the species from our dataset [72] (Fig. 5A, Table S7). *Pseudomonadota* appear to be predominantly aerobic and mapping the distribution of metabolism along the phylogeny showed no obvious trend (statistic Delta = 1.49, *P* value = 0.15 [74, 75]). Significant associations were observed when the type of metabolism was compared with the type of quinones (Fig. 5B, permutation test with 1000 iterations, *P* value <10^−3^, Methods S3): (i) species possessing only the UQ O_2_-dependent pathway have the highest proportion of aerobic metabolism; (ii) co-presence of the UQ O_2_-independent pathway increases the abundance of facultative metabolism (21.4% versus 6.6%); (iii) species with a low potential quinone in addition to UQ have a higher proportion of facultative metabolism (51.1%) and slightly higher proportion of anaerobic metabolism compared to species with UQ only (2.8% vs 0.4%). Overall, our data support that UQ is predominantly associated with aerobic metabolism and that the presence of low potential quinones allow for metabolisms with reduced dependence on O_2_, as organisms with low potential quinones display in proportion more anaerobes (permutation test, *P* value <10^−3^, Methods S3, Table S7).

### Integrative evolutionary scenario of quinone pathways in *Pseudomonadota*

**Figure 6:**
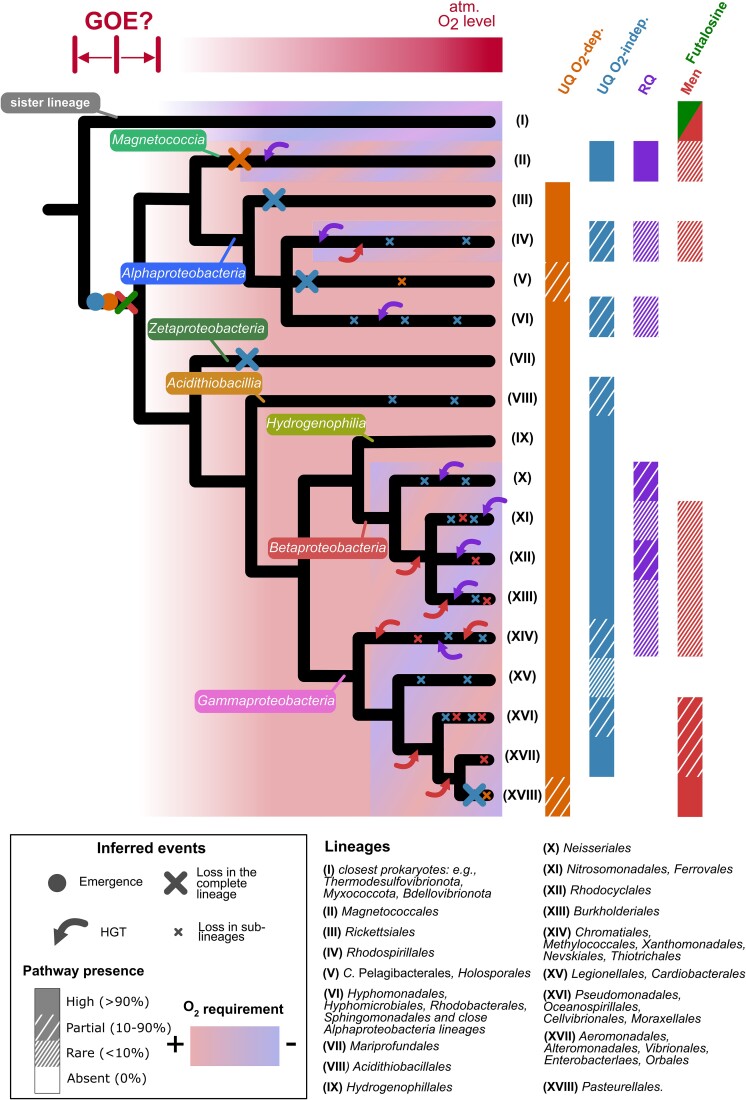
**Global evolutionary scenario for the quinone repertoire of *Pseudomonadota*.** The species tree of *Pseudomonadota*, made from a consensus of previous studies [3,5,86,100,101], is shown as a schematic of the *Pseudomonadota* phylogeny, which is currently not completely resolved. Closely related lineages are grouped together in a same branch whereas lineages with noteworthy compositions are distinguished in the tree with roman numbers opposite to the leaves. The degree of presence of each pathway in the genomes was categorized in four levels: High (>90% of the genomes), partial (between 10% and 90%), rare (<10%), absent, and is symbolized with different densities of hatchings. The emergence of the O_2_-independent and O_2_-dependent pathways in the common ancestor of *Pseudomonadota* is represented respectively by the blue and orange circles. Pathway losses are represented by large crosses when they cover the entire lineage(s), or by small crosses when they correspond to a partial loss from the lineage(s). The red and green cross in the ancestor of *Pseudomonadota* represents the loss of an ancestral MK pathway (either futalosine or men, see main text). Horizontal transfer events are represented by curved arrows.

Taken together, our data suggest the following evolutionary scenario to explain the distribution of quinones in *Pseudomonadota*. We propose that (i) MK biosynthesis (either via the futalosine or the Men pathways that are both present in sister lineages) was lost in a common ancestor of the *Pseudomonadota* (Fig. 6); (ii) UQ biosynthesis was an innovation of a common ancestor of the *Pseudomonadota*. Indeed, UQ pathways are absent from other prokaryotes and phylogenies of Ubi proteins strongly suggest their vertical transmission in *Pseudomonadota*. Proteins from the UQ O_2_-independent pathway (*ubiTUV*) and proteins from the UQ O_2_-dependent pathway (O_2_-dependent hydroxylases) [40] all appear ancestral, but the relative timing of emergence of the two pathways cannot be retraced due to insufficient evidence. The O_2_-dependent pathway has undergone very few loss events, except for the *Pasteurellales* (Fig. 1A, lineage XVIII in Fig. 6), whereas the absence of the O_2_-independent pathway in many lineages leads us to conclude that *ubiTUV* genes have been repeatedly lost, resulting in a complete absence in several lineages (lineages III, V, VII, XVIII in Fig. 6) and a partial presence in others (IV, VI, VIII, XIV-XVII in Fig. 6). (iii) Low potential quinones (either MK or RQ) were acquired secondarily in several lineages of *Pseudomonadota* and probably allowed the diversification of metabolisms with reduced dependence on O_2_. We propose that RQ biosynthesis originated in *Pseudomonadota* because they have UQ available as a substrate for RquA and they contain SAM-dependent methyltransferase homologs that could have diversified into RquA [78,99]. *rquA* then probably spread to multiple lineages through a combination of vertical transmission and HGT. MK production was acquired secondarily by *Pseudomonadota* and we could document at least seven HGT events of the Men pathway from distinct bacterial lineages (Fig. 6). The Men pathway is localized at a single genetic locus in extant *Pseudomonadota* (Fig. 4B) and other bacterial lineages, which probably facilitates the transfer of the entire pathway in a single event. Scattered occurrences of the Men pathway in several *Pseudomonadota* lineages (lineages IV and XI-XIV in Fig. 6) could be explained by repeated losses. The Men pathway is highly conserved with the UQ O_2_-independent pathway in *Gammaproteobacteria* species that are predominantly facultative aerobes (Fig. 5A, lineages XVI-XVII in Fig. 6), suggesting complementary functions for UQ and MK under low O_2_ conditions. A closer investigation of the phylogenies of these lineages suggests that the Men pathway was predominantly acquired by ancestral species that had the UQ O_2_-independent pathway (Fig. S2).

## Discussion

A striking feature of the proposed evolutionary scenario is the dynamics of the low potential quinones repertoire. This scenario proposes that a low potential quinone (MK) was lost in an ancestor of *Pseudomonadota*, while a high potential quinone (UQ) emerged and played a pivotal role in enabling adaptation to oxic environments. Neither the nature of the ancestral MK pathway nor the timing of its loss could be inferred precisely, but our analyses clearly indicate that MK was lost early in the diversification of *Pseudomonadota*. It is therefore striking that low potential quinones were secondarily reacquired by several lineages, as evidenced by multiple gains of the Men pathway by HGT (Figs. 4, 6, S13). We found these cases in lineages with a very high proportion of organisms possessing the O_2_-independent UQ pathway (Fig. 1B-C), suggesting that the presence of this ancestral pathway facilitates the reacquisition of the Men pathway. One could extrapolate that species with the O_2_-independent UQ pathway may have had more opportunities to acquire the Men pathway, as they may have been able to cope with lower O_2_ concentrations, thus favouring encounters with (facultative) anaerobic organisms possessing the Men pathway. The conserved genetic proximity of Men pathway genes (Figs. 4B, S9) most likely further favoured their horizontal acquisitions. Furthermore, lineages possessing the Men pathway correspond to those with higher proportions of facultative aerobes/anaerobes (Fig. 5B). Taken together, this suggests that after extensive diversification into oxic environments, as evidenced by the widespread presence of heme-copper oxygen reductase (Fig. 5A), several *Pseudomonadota* lineages “returned” to conquer less oxic environments. This was possible thanks to the conservation of the O_2_-independent UQ pathway and the acquisition of a MK (or RQ) pathway, which allowed the expansion of anaerobic respiration capacities by providing access to a different palette of dehydrogenases and reductases in the ETC.

A compelling case for quinone dynamics is that of the *Pasteurellales* (lineage XVIII, Fig. 6), a lineage belonging to a subgroup of *Gammaproteobacteria* that acquired the Men pathway (subgroup made of lineages XVII-XVIII, Fig. 6). Our data support that the acquisition of MK was followed by the loss of the ancestral UQ pathway in some species of *Pasteurellales* (Table S1). *Pasteurellales* are facultative anaerobes capable of switching to fermentative metabolism and include mostly commensals and opportunistic pathogens of vertebrates that generally poorly survive outside their hosts (e.g., *Haemophilus influenzae*, *Pasteurella multocida*, *Aggregatibacter actinomycetemcomitans*) [102]. In contrast to other lineages of *Pseudomonadota*, most *Pasteurellales* do not possess heme-copper oxygen reductase (most of them have a low affinity for O_2_), but many possess candidate high-affinity Cyt *bd* oxidases that are more adapted to microaerobic conditions (Fig. 5A, Table S6). We hypothesize that *Pasteurellales* are undergoing specialization to their host niches, progressively losing the use of a high potential quinone as they rely more and more on the low potential MK (and demethyl-MK). In *Pasteurellales*, as well as in other lineages that have acquired MK (or RQ), it would be worth investigating whether the acquisition of the low potential quinone was accompanied by the acquisition of other genes. For example, one might expect a gain of the fumarate reductase, which is part of the anaerobic ETC and functions with low potential quinones, whereas the related succinate dehydrogenase is part of the aerobic ETC and functions with UQ [64,103]. Alternatively, some proteins may be intrinsically capable or could have evolved the ability to function with both UQ and MK, as demonstrated for *E. coli* nitrate reductase A (NarGHI), which accommodates demethyl-MK, MK and UQ [104,105]. More generally, the entire ancestral bioenergetic metabolism of *Pseudomonadota* must have adapted to the use of high potential quinones in the first place [27,106], which implies the adaptation of the repertoire of dehydrogenases and reductases either through the recruitment of novel enzymes by HGT or through the adaptation of pre-existing enzymes. Studying the dynamic repertoire of respiratory quinones enables the identification of the most interesting lineages to further explore the multiple energy metabolism transitions that *Pseudomonadota* have undergone during their evolution.

From our results, it is clear that the capacity to produce the high potential UQ was acquired before the diversification of extant *Pseudomonadota*. However, we could not determine which of the O_2_-dependent or O_2_-independent pathways appeared first. Indeed, we showed that both pathways are vertically transmitted and are globally present in all classes of *Pseudomonadota*, except for the *Magnetococcia*, which possess only the O_2_-independent pathway (Fig. S7, Table S5D). Because *Magnetococcia* appears as the sister group of *Alphaproteobacteria* in phylogenies of *Pseudomonadota* (Fig. 3) [85,87–89], we infer that the O_2_-independent and O_2_-dependent pathways were both present in the last common ancestor of *Pseudomonadota* (Fig. 6), and that the *Magnetococcia* lost the O_2_-dependent pathway to retain only the O_2_-independent version, in line with the adaptation of *M. marinus* to microoxic niches [70]. The two pathways were necessarily acquired sequentially in the ancestor of *Pseudomonadota* and the future discovery of deep-branching lineages of *Pseudomonadota* could provide more resolution to determine which UQ pathway appeared first. If *Pseudomonadota* emerged before the GOE, as proposed by several studies [107,108], the acquisition of the O_2_-independent pathway first is an attractive hypothesis. O_2_-dependent hydroxylases would have been co-opted later in the UQ pathway, in line with what is proposed for the bacteriochlorophyll or cobalamin pathways for which O_2_-independent versions appeared first and O_2_-dependent versions followed later [109]. The observation that the genes specific to the O_2_-independent pathway are sometimes encoded next to *ubi* genes from the common part of the pathway (Figs. S7, S8), whereas this is never observed for the genes specific to the O_2_-dependent pathway, also favours the hypothesis that the O_2_-independent version evolved first.

To date, only three prokaryotic lineages are known to synthesize high potential quinones: the oxygenic *Cyanobacteria* with plastoquinone, the *Pseudomonadota* with UQ, and the archaea of the order *Sulfolobales* with caldariellaquinone and sulfolobusquinone [29, 30, 36]. This narrow distribution may in part be due to the difficulty of acquiring high potential quinones via HGT, as the UQ and plastoquinone pathways are now encoded at multiple genetic loci (Figs. S8, S9) [110], unlike the Men pathway for which we documented several cases of transfer. The plastoquinone and UQ pathways are evolutionarily related and share several homologous enzymes [110–112]. These high potential quinones could have evolved independently from an ancestral biosynthetic pathway, or one lineage could have acquired its high potential quinone from the other [112]. Investigating the relative origins of the UQ and plastoquinone pathways may help determine which of the two high potential quinones evolved first [111]. This could also provide a new angle to link the origin and diversification of *Pseudomonadota* to the GOE, as oxygenic photosynthesis responsible for the rise of O_2_ levels on Earth, appeared in Cyanobacteria. Because the hydroxylation step of the extant cyanobacterial plastoquinone pathway has been proposed to involve a candidate O_2_-dependent hydroxylase [111], we note that ancestral Cyanobacteria may have produced plastoquinone via an O_2_-independent pathway or may have used a quinone other than plastoquinone to evolve oxygenic photosynthesis. In any case, the existence of the O_2_-independent UQ pathway opens the possibility of the appearance of a high potential quinone before the GOE. Integrating our results with a thorough study of the evolution of the quinone repertoire and of their biosynthetic pathways in *Cyanobacteria* and *Sulfolobales* may shed light on how and when high potential ETC first appeared, and how organisms adapted their energy metabolism to new quinones and increasing O_2_ levels.

## Supplementary Material

TableS1-annotation_pathways_reviews_wrae253

TableS2-RQ_exp_results_wrae253

TableS3-TUV_table_wrae253

TableS4-fnr_triplets_wrae253

TableS5-annotation_extra_genomes_wrae253

TableS6-annotation_reductases_wrae253

TableS7_metabolism_wrae253

Sup_Figs_revised_wrae253

Sup_Texts_revised_wrae253

## Data Availability

The data underlying this article are available in the article and in its online supplementary material. In addition, datasets S1 and S4 are available at the Figshare repository and can be accessed at https://doi.org/10.6084/m9.figshare.26390974 for Dataset S1 and at https://doi.org/10.6084/m9.figshare.26391025 for Dataset S4.
