# Supplementary material for: Dynamic quinone repertoire accompanied the diversification of energy metabolism in Pseudomonadota
Source: ISME J. 2024 Dec 18;19(1):wrae253. doi: 10.1093/ismejo/wrae253 (PMC11707229; doi:10.1093/ismejo/wrae253)
Supplement: Sup_Figs_revised_wrae253 [file sup_figs_revised_wrae253.pdf]

Supplementary Figures for:

Dynamic quinone repertoire accompanied the diversification of energy metabolism in Pseudomonadota

Sophie-Carole Chobert, Morgane Roger-Margueritat, Laura Flandrin, Safa Berraies, Christopher T. Lefèvre, Ludovic Pelosi, Ivan Junier, Nelle Varoquaux, Fabien Pierrel, Sophie S. Abby

Fig. S1 UQ, RQ and (D)MK biosynthetic pathways in prokaryotes
Fig. S2 *Gammaproteobacteria* species tree and the Men pathway
Fig. S3 UQ pathway genes contiguity in different *Pseudomonadota* classes
Fig. S4 Synteny matrix of genes belonging to the Men, UQ and RQ pathways in *Pseudomonadota*
Fig. S5 Covariance matrices of Mqn, Men, Ubi and RquA proteins in prokaryotes
Fig. S6 Phylogeny and experimental characterization of RquA
Fig. S7 Phylogeny of RquA
Fig. S8 Assessing the presence of a pathway based on the distribution of the number of different proteins for each quinone pathway in genomes
Fig. S9 Distance of candidate FNR sites from the start codon of *ubiT* or *ubiU* for each main genetic architecture
Fig. S10 Characterisation of the quinone content of *Magnetococcus marinus*
Fig. S11 Genetic organisation of two Magnetococcia UQ pathways and their genetic environment (-5/+5 genes)
Fig. S12 Presence of quinone pathways genes at the order level in *Pseudomonadota*
Fig. S13 Phylogenies of core genes of UQ pathways
Fig. S14 Rooted phylogenies of UQ proteins: UbiV, -U, -A and -D
Fig. S15 Phylogeny of MenF
Fig. S16 Automatic reconciliation of the MenF, MenD and MenB phylogenies with the *Pseudomonadota* species tree.

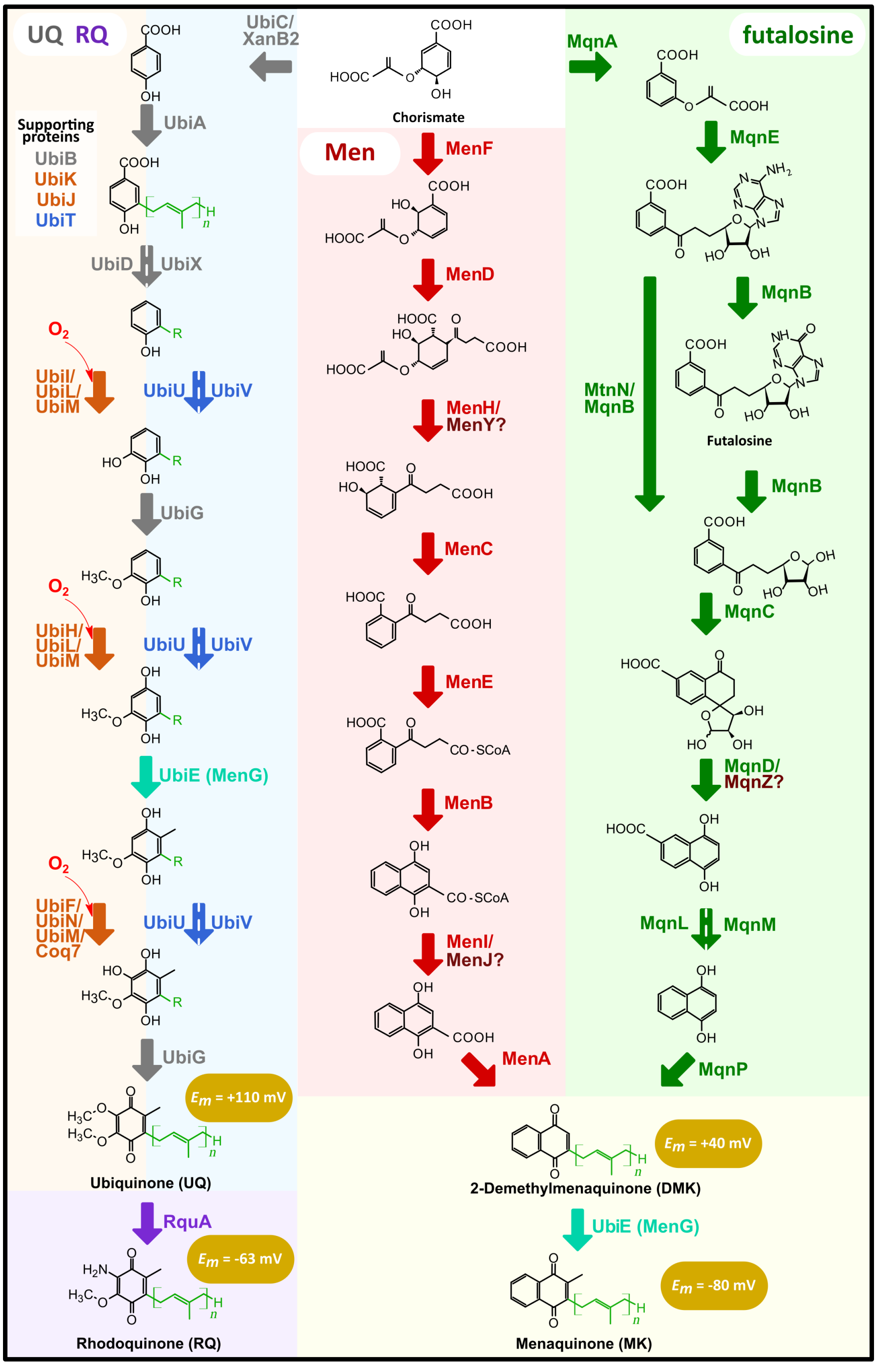

Fig. S1 UQ, RQ and (D)MK biosynthetic pathways in prokaryotes. Reactions are depicted by the wide coloured arrows with protein names specified in gray and blue for the UQ pathway, violet for the RQ pathway, red for the Men MK pathway and green for the futalosine MK pathway. For the UQ pathway, the requirement of O_2 in the hydroxylation steps of the O_2-dependent pathway (large orange arrows) is depicted by thin red arrows. The proteins participating specifically in the O_2-independent pathway are shown in blue, whereas proteins participating in both the O_2-dependent and O_2-independent UQ pathways (common part) are displayed in gray. Other non-enzymatic proteins are indicated as "Supporting proteins". UbiE (also called MenG) is displayed in cyan as it contributes to both the UQ and MK biosynthetic pathways. The redox potential (E_m) of the quinone produced by each pathway is specified. A dashed white line is drawn when two enzymes jointly perform a reaction. If a step can be made by alternative enzymes, then their names are separated by a forward slash. The names of uncertain enzymes are indicated in burgundy with a question mark. We note that the order of the decarboxylation (MqnL, MqnM) and prenylation (MqnP) remains speculative [1]. Biosynthetic intermediates are shown in the reduced state whereas UQ, RQ and (D)MK are represented in their oxidised state, resulting from a two electrons oxidation (not shown).

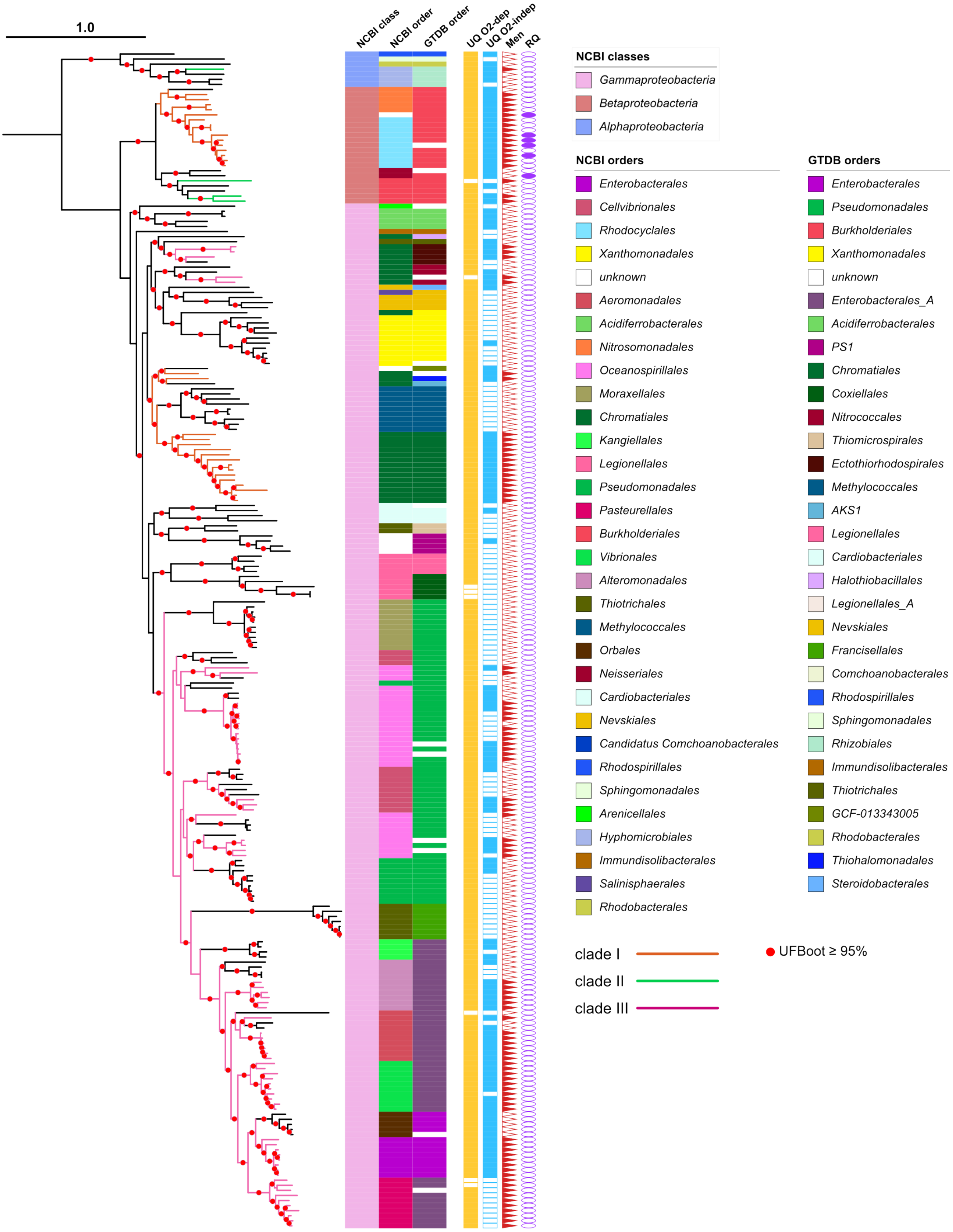

Fig. S2 *Gammaproteobacteria* species tree and the Men pathway. The tree is made from the concatenation of 41 marker genes of a sampling of *Gammaproteobacteria* and was rooted using an outgroup of *Alphaproteobacteria* and *Betaproteobacteria* sequences. The tree branches are colored whenever the Men pathway is present. The 3 colors correspond to the 3 clades described in the main text (Fig. 4). The tree displays the class-level NCBI taxonomy, order-level NCBI taxonomy, order-level GTDB taxonomy and the presence of the UQ, Men and RQ pathways. The tree scale bar expresses the number of substitutions per site.

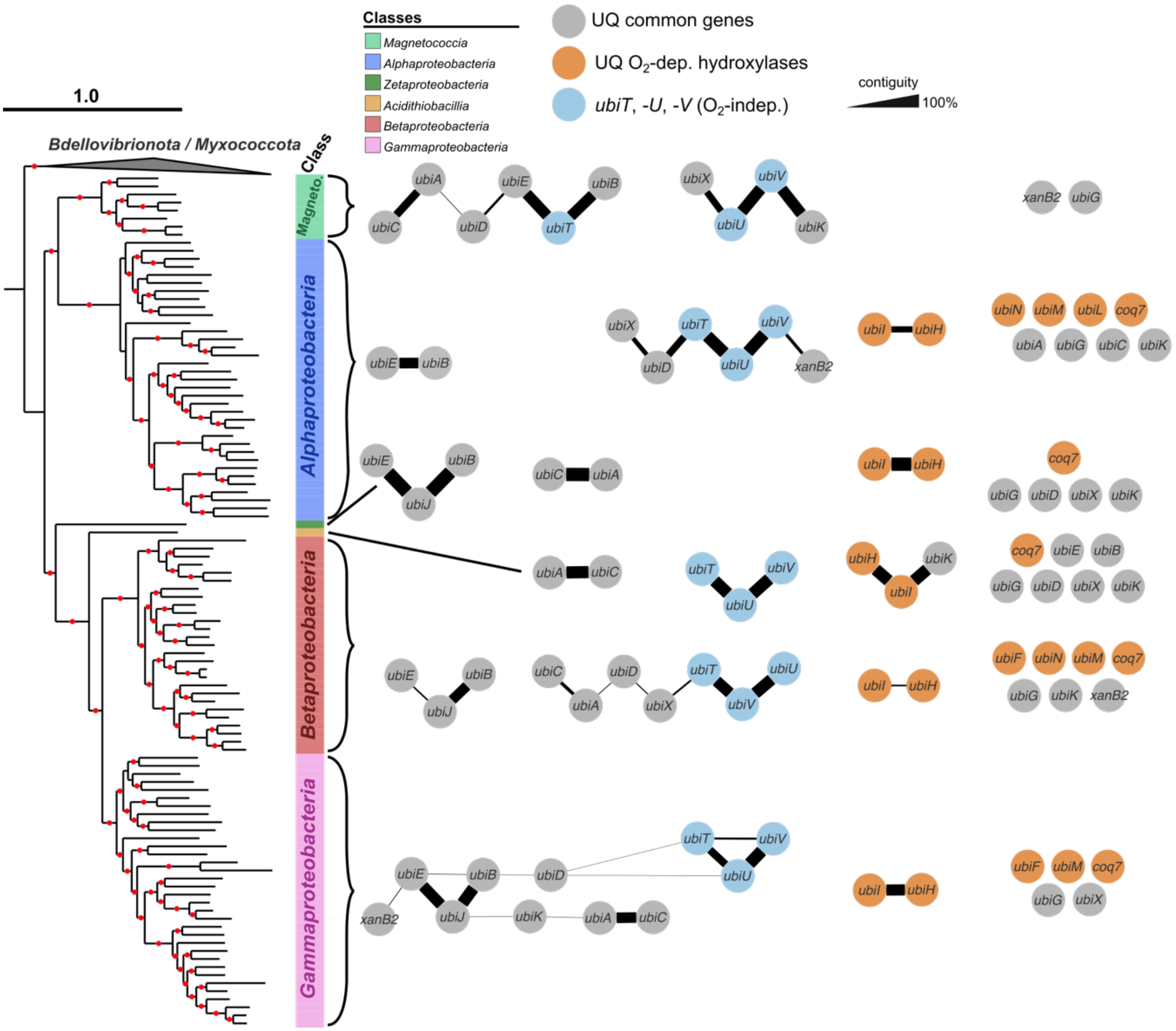

Fig. S3 UQ pathway genes contiguity in different *Pseudomonadota* classes. Opposite the species tree (see Fig. 3) is the taxonomy at class-level, followed by the gene contiguity graphs. When there is a line between two genes, these two genes have been seen side by side at least once. The thickness of the line corresponds to the percentage of contiguity for each pair (number of times adjacent divided by the least present of the two genes). Apart from the gene sets *ubiE-J/T-B*, *ubiA-C*, *ubiT-U-V,* and *ubiH-I*, the other genes involved in UQ production do not show strong contiguity. Note that mis-annotated UbiL in *Francisella* were reassigned as UbiH or UbiI according to the phylogeny from [2]. The branches with high support (UFBoot ≥ 95%) are indicated by red dots. Scale bar of the tree is expressed in substitutions per site.

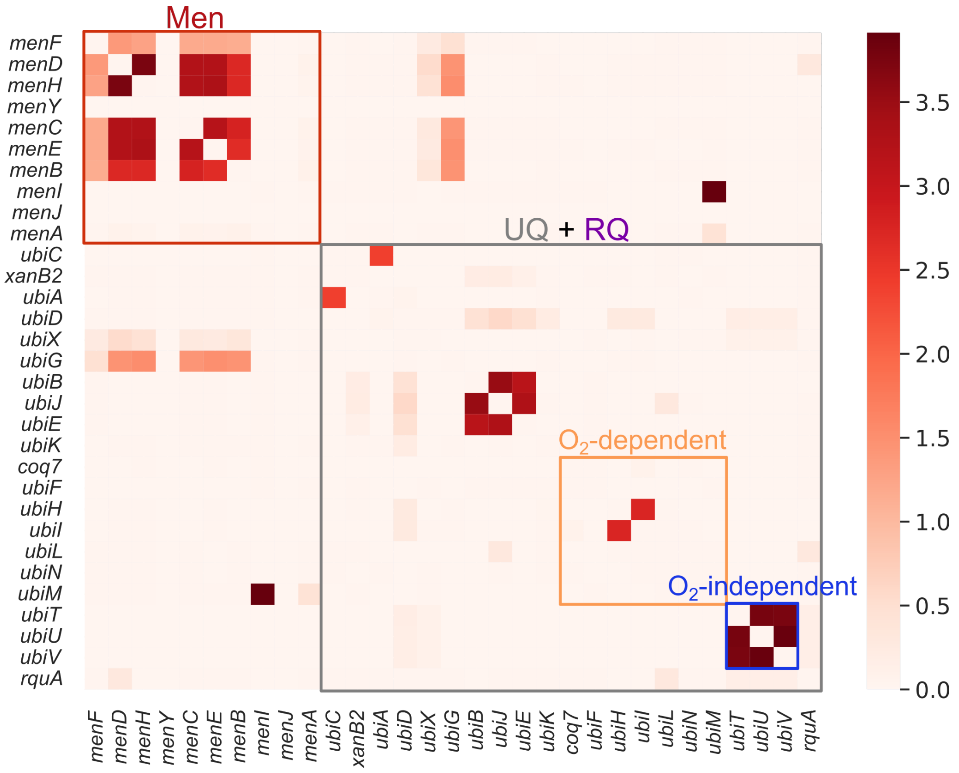

Fig. S4 Synteny matrix of genes belonging to the Men, UQ and RQ pathways in *Pseudomonadota*. Genes are grouped by pathways in genomes. This matrix was obtained by considering only one version of each gene (when there are >1 hit, we chose the one with the highest GA score) per genome in organisms with a predicted pathway. Synteny is measured by relative entropy which indicates how much the genes are colocalised as compared to random (see Materials and Methods). We observe an important synteny signal between genes that belong to the same pathway. Note that the strong signal between the genes *menI* and *ubiM* may not be relevant as it represents the synteny of the two genes in only one genome.

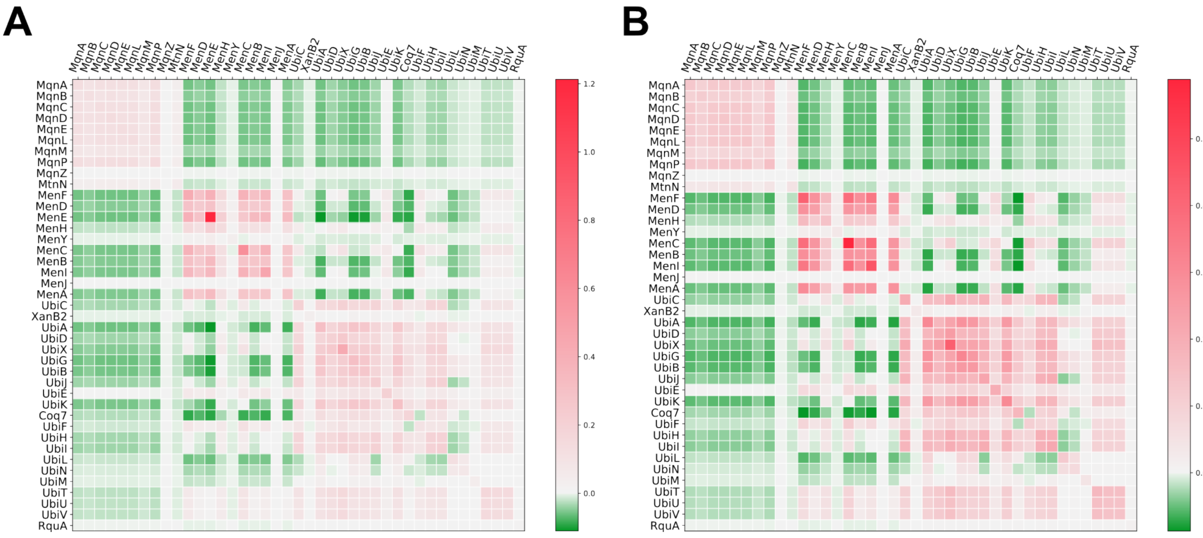

Fig. S5 Covariance matrices of Mqn, Men, Ubi and RquA proteins in prokaryotes. Proteins are gathered by pathway and they are ordered according to their known or assumed position in their biosynthetic pathway. **A** Matrix of the complete set of genes. **B** Matrix without MenE. Due to the lack of specificity of the MenE HMM profile, this protein appears overly annotated, leading to a flatten covariance matrix (matrix A).

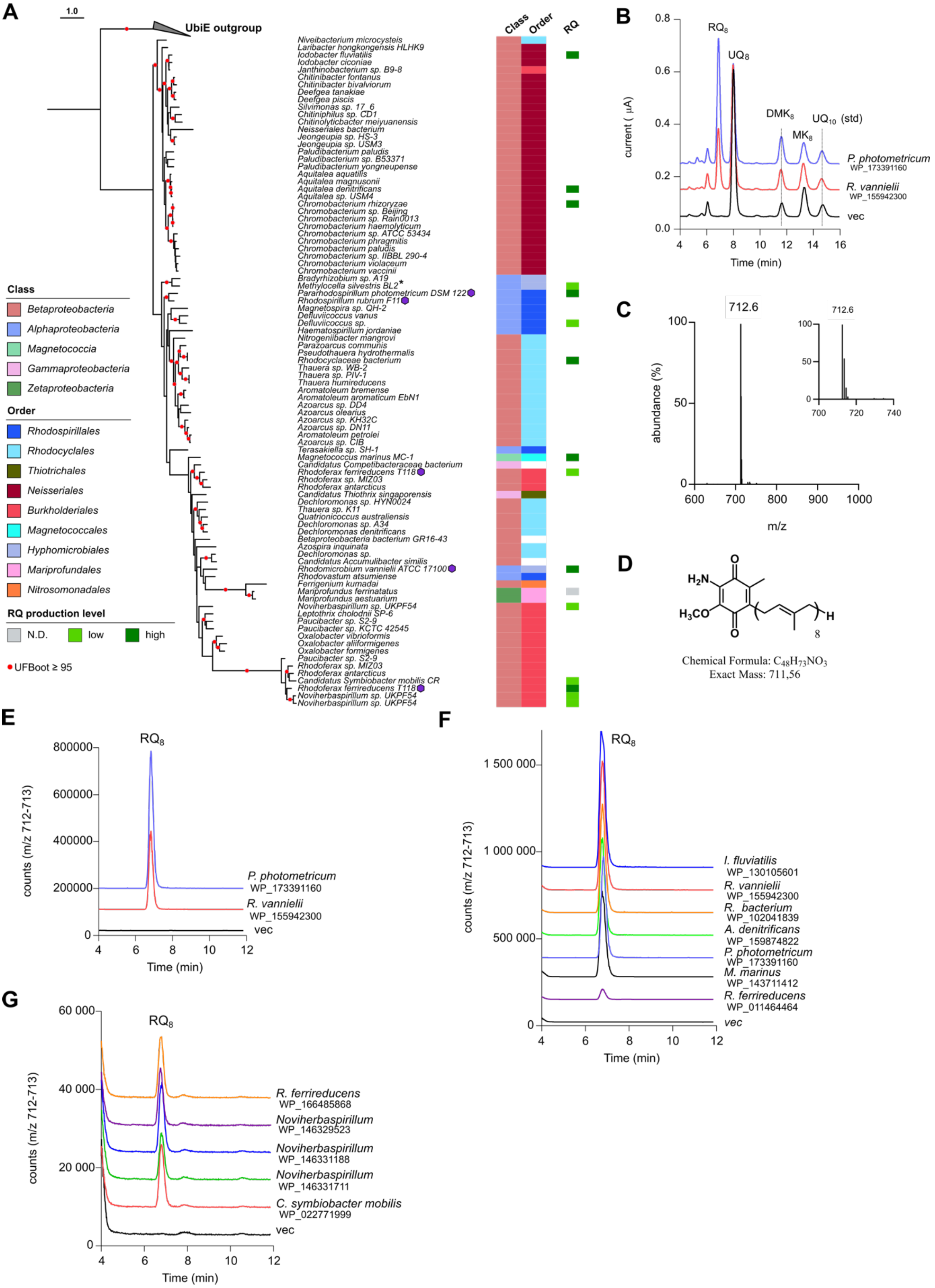

Fig. S6 Phylogeny and experimental characterisation of RquA. A Maximum likelihood phylogeny of RquA rooted with UbiE sequences as an outgroup. Despite being distantly related, UbiE sequences did not disturb the overall topology of the tree (Fig. S3). The tree is annotated with color strips at two taxonomic levels: class and order. The branches with high support (UFBoot ≥ 95%) are indicated by red dots. Species cited in the literature

as producing RQ are marked with a purple hexagon. *Methylocella silvestris* is lacking a predicted UQ pathway (*). The level of RQ production in the heterologous expression assay is represented with a three-color scale for the RquA sequences tested experimentally: gray indicates “N.D” (not detected), light green indicates a low production of RQ (<10 arbitrary units of RQ) and dark green indicates a high production of RQ (390 > x > 10 a.u. RQ) (see also Table S2 and Fig S2B-G). The tree was obtained from the analysis of 183 aligned positions using IQ-TREE with Q.pfam+I+I+R5 as the best selected model. The tree scale bar expresses the number of substitutions per site. **B** Overlay of HPLC-electrochemical detection analyses of lipid extracts from *E. coli* cells containing an empty plasmid (vec) or plasmids with RquA homologs. UQ_8, DMK_8 and MK_8 are natural quinones produced by *E. coli* and RQ_8 is produced only in cells expressing RquA homologs. UQ_{10} was used as an internal standard. **C** Mass spectrum of the compound eluting at 6.8 min in the HPLC analysis of *E. coli* cells with the plasmid containing the RquA homolog from *P. photometricum* (see Fig. S2B). The isotope mass is shown as inset. **D** Chemical formula of RQ_8. **E-G** Superimposition of chromatograms obtained by single ion monitoring (m/z 712-713 corresponding to RQ_8+H^+) of lipid extracts from *E. coli* cells expressing the indicated RquA homologs or containing the empty plasmid (vec). The chromatograms are representative of at least three independent samples (B, E-G). The baseline is 0 for all chromatograms, but has been arbitrarily shifted for visualisation (B, E-G).

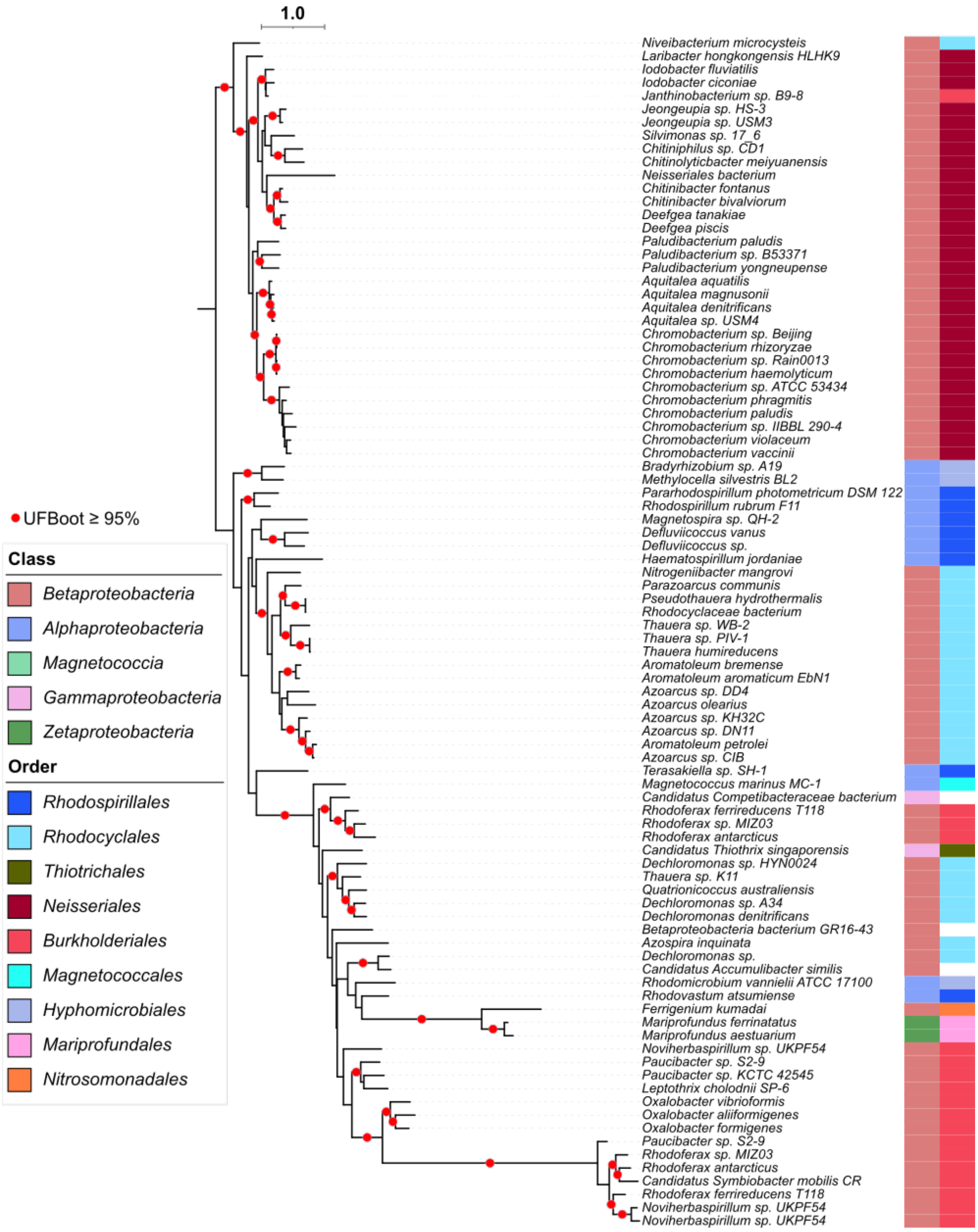

Fig. S7 Phylogeny of RquA. A maximum likelihood phylogeny of RquA is presented. The tree is annotated with colored stripes at two taxonomic levels: class and order. The branches with high support (UFBoot ≥ 95%) are indicated by red dots. The tree was obtained from the analysis of 281 aligned positions using IQ-TREE with Q.pfam+F+R5 as the best selected model. The tree scale bar expresses the number of substitutions per site.

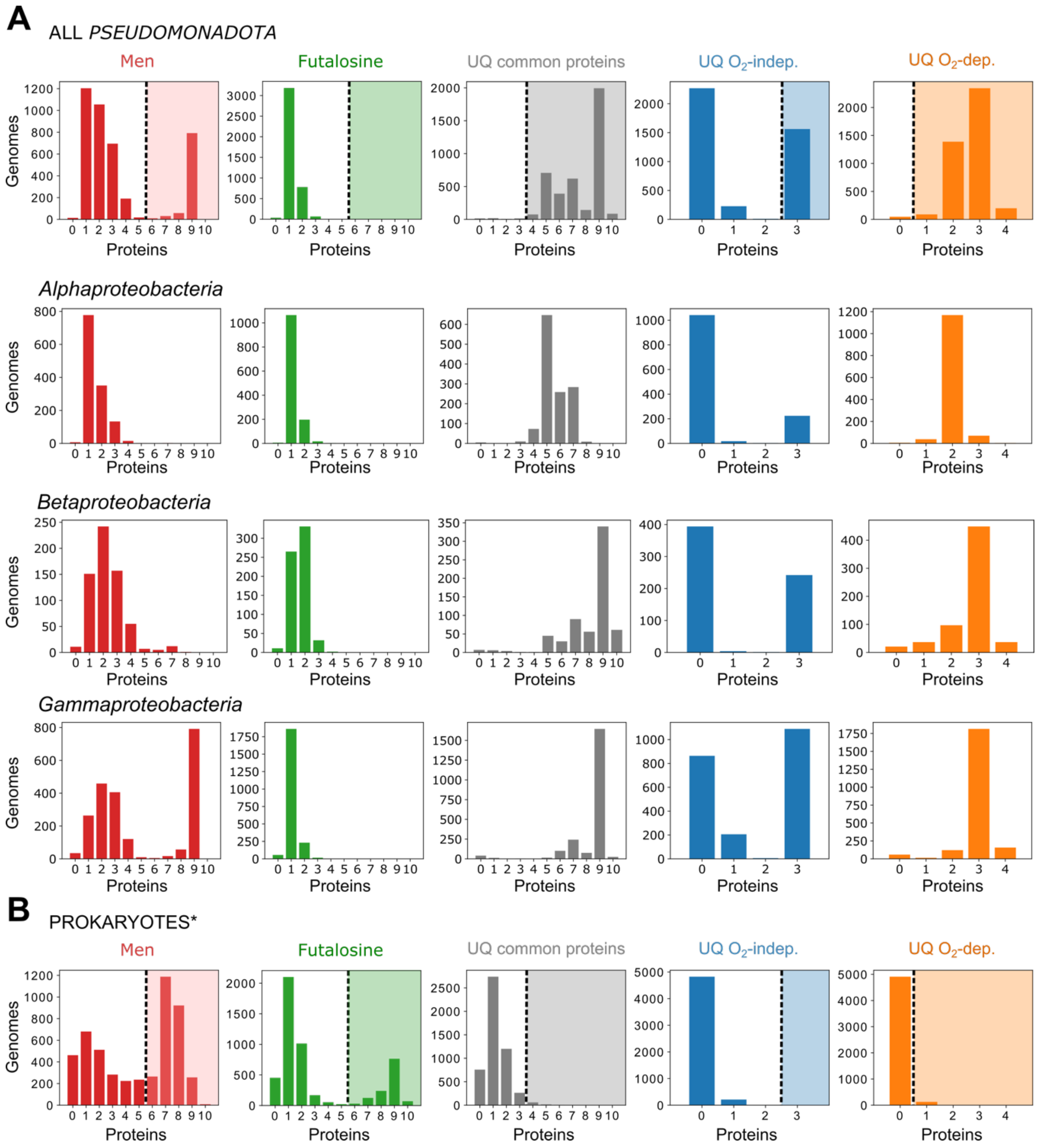

Fig. S8 Assessing the presence of a pathway based on the distribution of the number of different proteins for each quinone pathway in genomes. HMM profiles were designed for all the proteins involved in quinone pathways: the Men MK pathway, the futalosine MK pathway, proteins common between the two UQ pathways and proteins involved only in the O_2-independent (UbiT, -U, -V) and the O_2-dependent (UbiF, -H, -I, -L, -M, -N, Coq7) pathways. **A** Distributions of the number of positive hits for HMM profiles of each pathway in 4107 *Pseudomonadota* genomes and in the three major classes of *Pseudomonadota*. **B** Distributions obtained with 5392 genomes from prokaryotes excluding *Pseudomonadota*. Dashed lines represent the threshold used as the minimal number of proteins required for the inference of the pathway, while the colored area represent the genomes for which the pathway is therefore inferred to be present.

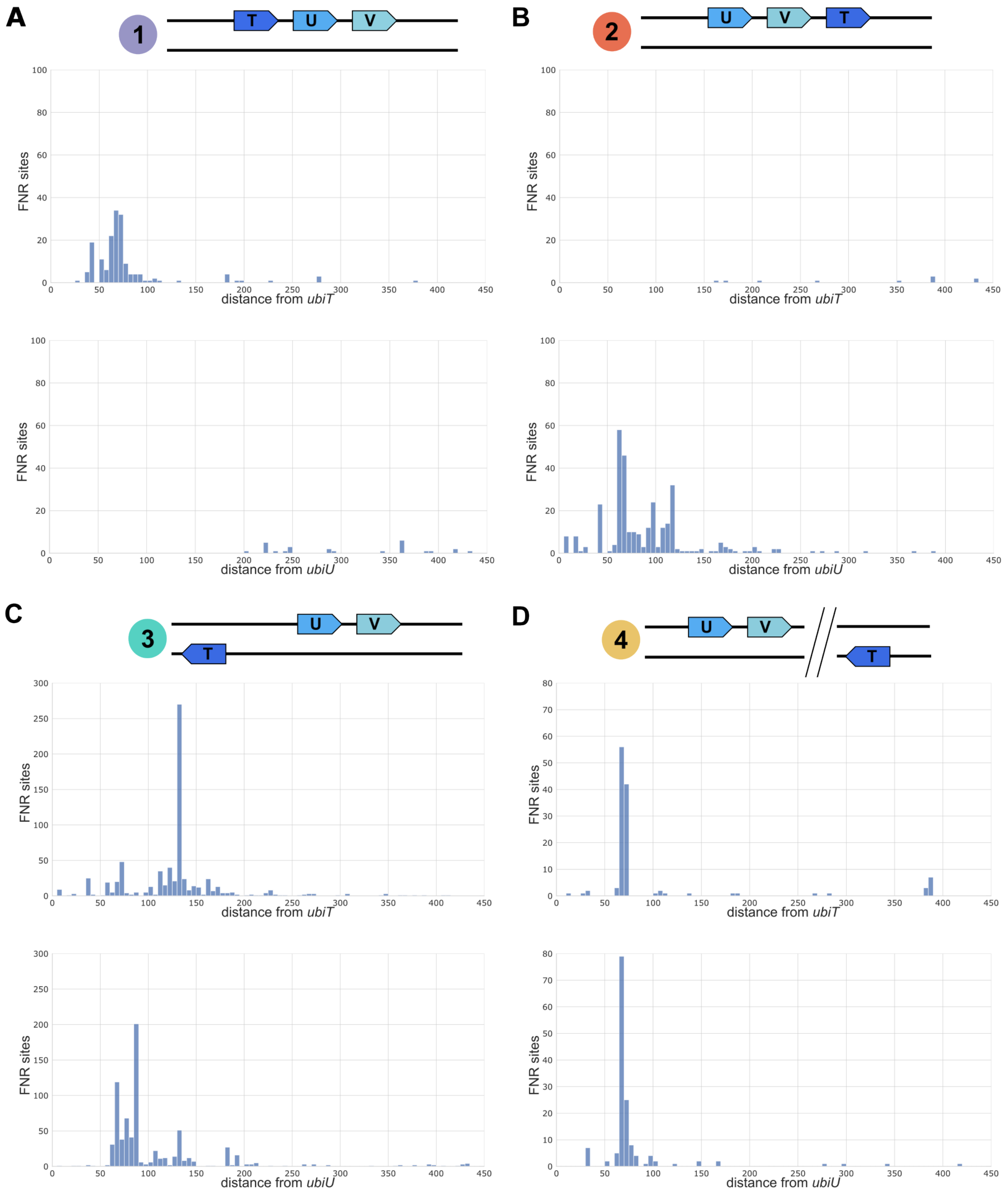

Fig. S9 Distance of candidate FNR sites from the start codon of *ubiT* or *ubiU* for each main genetic architecture. Each letter corresponds to the architectures from 1 to 4 described in Fig. 2B. For each architecture the distance distribution of all FNR sites predicted are represented relative to *ubiT* (top lines), or to *ubiU* (bottom lines).

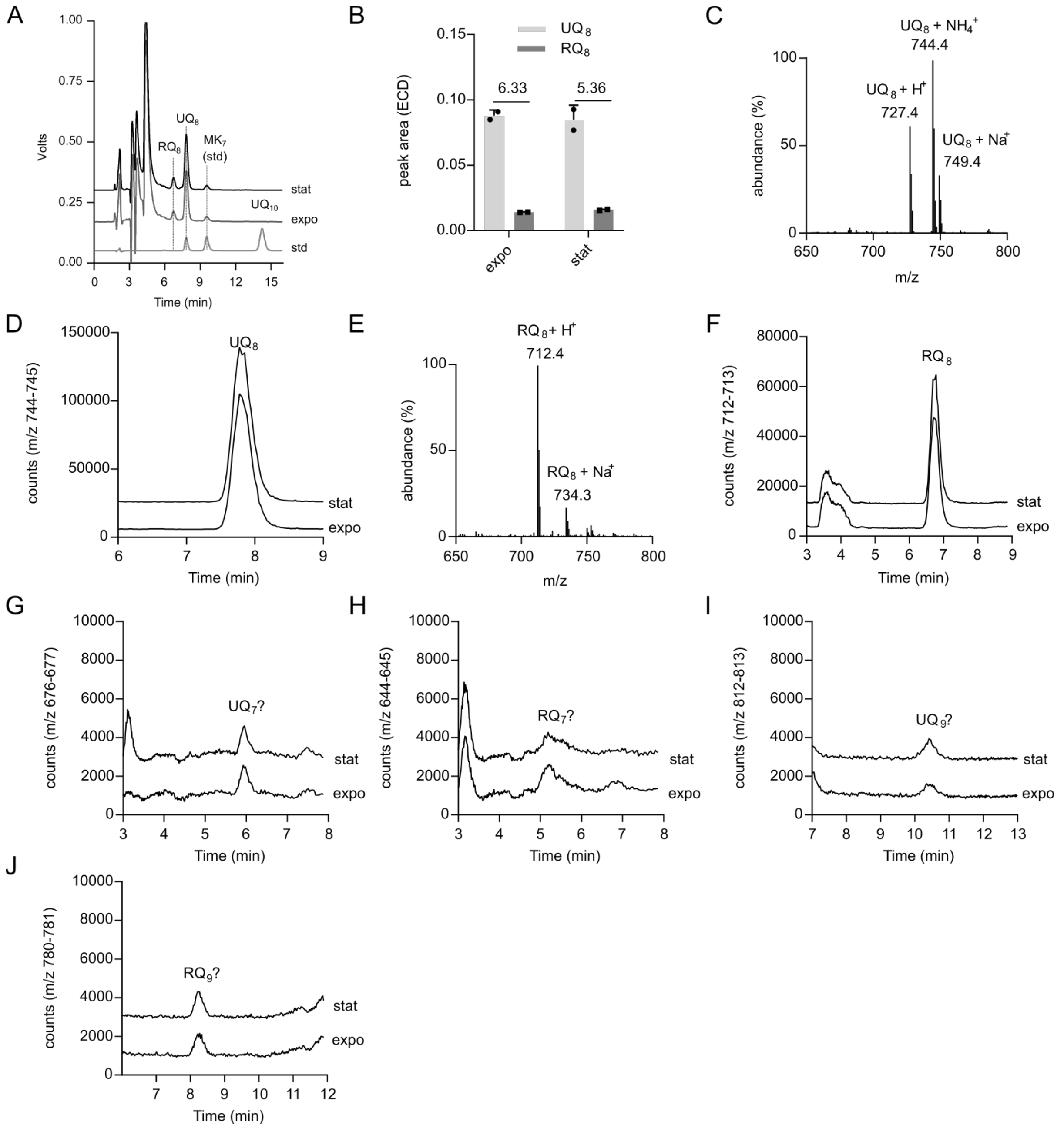

Fig. S10 Characterisation of the quinone content of *Magnetococcus marinus*. **A** Overlay of HPLC-electrochemical detection analyses of lipid extracts from $5*10^7$ *M. marinus* strain MC-1 cells grown either to exponential phase ("expo") or to stationary phase ("stat"). A standard ("std") containing UQ_8, MK_7 and UQ_{10} was analysed in the same conditions and MK_7 was added to cells as an internal standard prior to organic extraction (see Materials and Methods). **B** Comparison of the integration values of ECD peaks corresponding to UQ_8 and RQ_8 in extracts from cells grown to exponential phase (expo) or to stationary phase (stat). Bars represent means ± SD (n=2) and values indicate the mean ratio between UQ_8 and RQ_8. **C** Mass spectrum of UQ_8 eluting at 7.8 min in extracts of *M. marinus* cells (A) showing that $UQ_8 + NH_4^+$ is the main adduct detected at m/z 744.4. **D** Single ion monitoring for $UQ_8+NH_4^+$ (m/z 744-745) in extracts of *M. marinus* cells. **E** Mass spectrum of RQ_8 eluting at 6.8 min in extracts of *M. marinus* cells (A). **F** Single ion monitoring for RQ_8+H^+ (m/z 712-713) in extracts of *M. marinus* cells. **G-J** Single ion monitoring for $UQ_7+NH_4^+$ (m/z 676-677, G), $UQ_9+NH_4^+$ (m/z 812-813, I), RQ_7+H^+ (m/z 644-645, H) and RQ_9+H^+ (m/z 780-781, J). Given the weak intensity of the peaks, the identity of the compounds remains hypothetical (G-J). The data shown are representative of biological duplicates (A-J). The baseline is 0 for all chromatograms (A, D, F-J), but has been arbitrarily shifted to help visualisation.

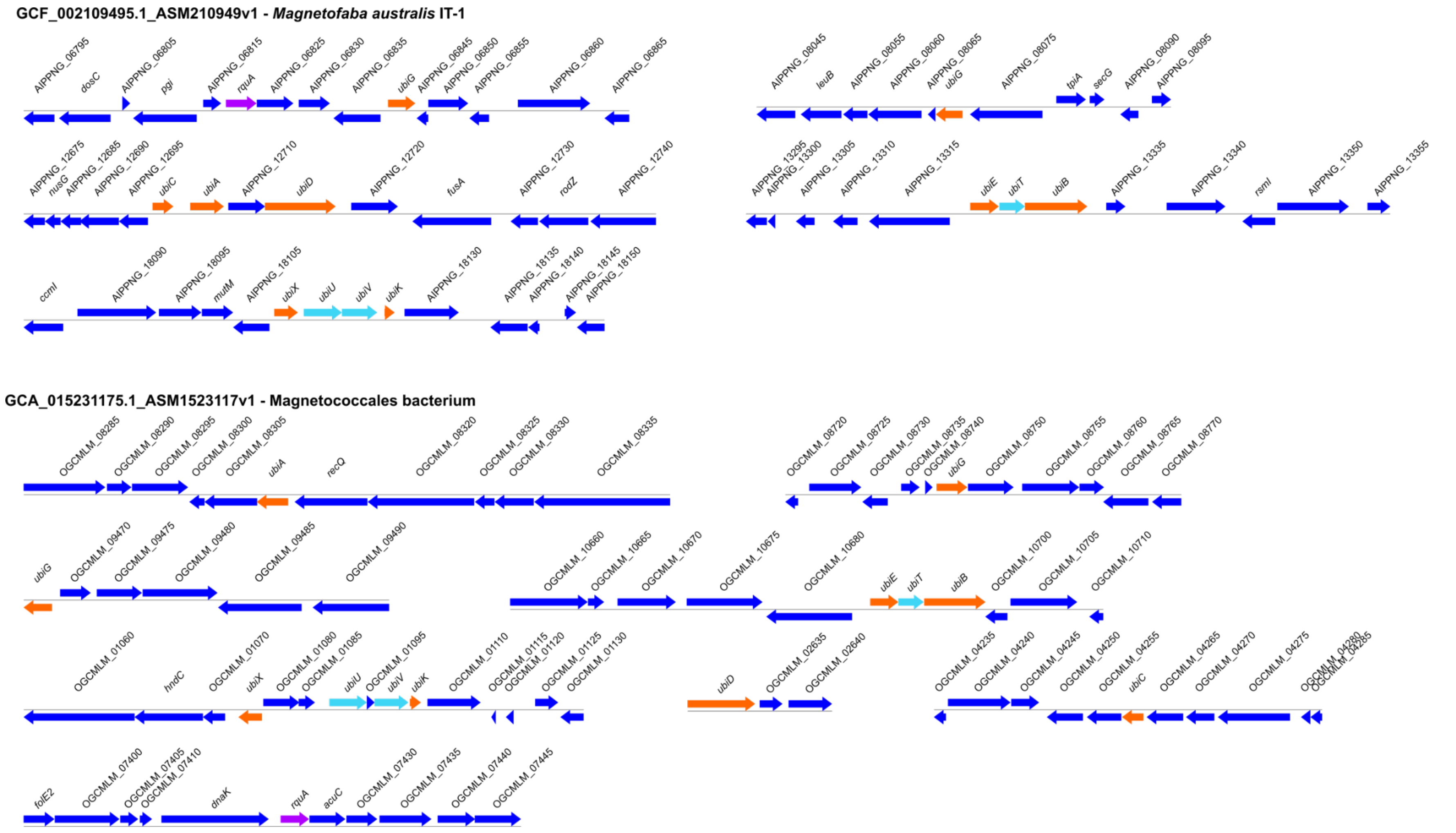

Fig. S11 Genetic organisation of two Magnetococcia UQ pathways and their genetic environment (-5/+5 genes). The *ubi* genes from the core UQ pathway are colored in orange; *ubiT, -U* and *-V* genes are colored in light blue; *rquA* is in purple; and other genes are in dark blue. Gene annotation is in order of priority: (i) our annotation, (ii) the gene name predicted in the GFF files generated by bakta, (iii) the CDS identifier generated by bakta. Arrows type sketches were drawn using the GenomeViz Python library (v0.4.4) in a home-made script (https://moshi4.github.io/pyGenomeViz).

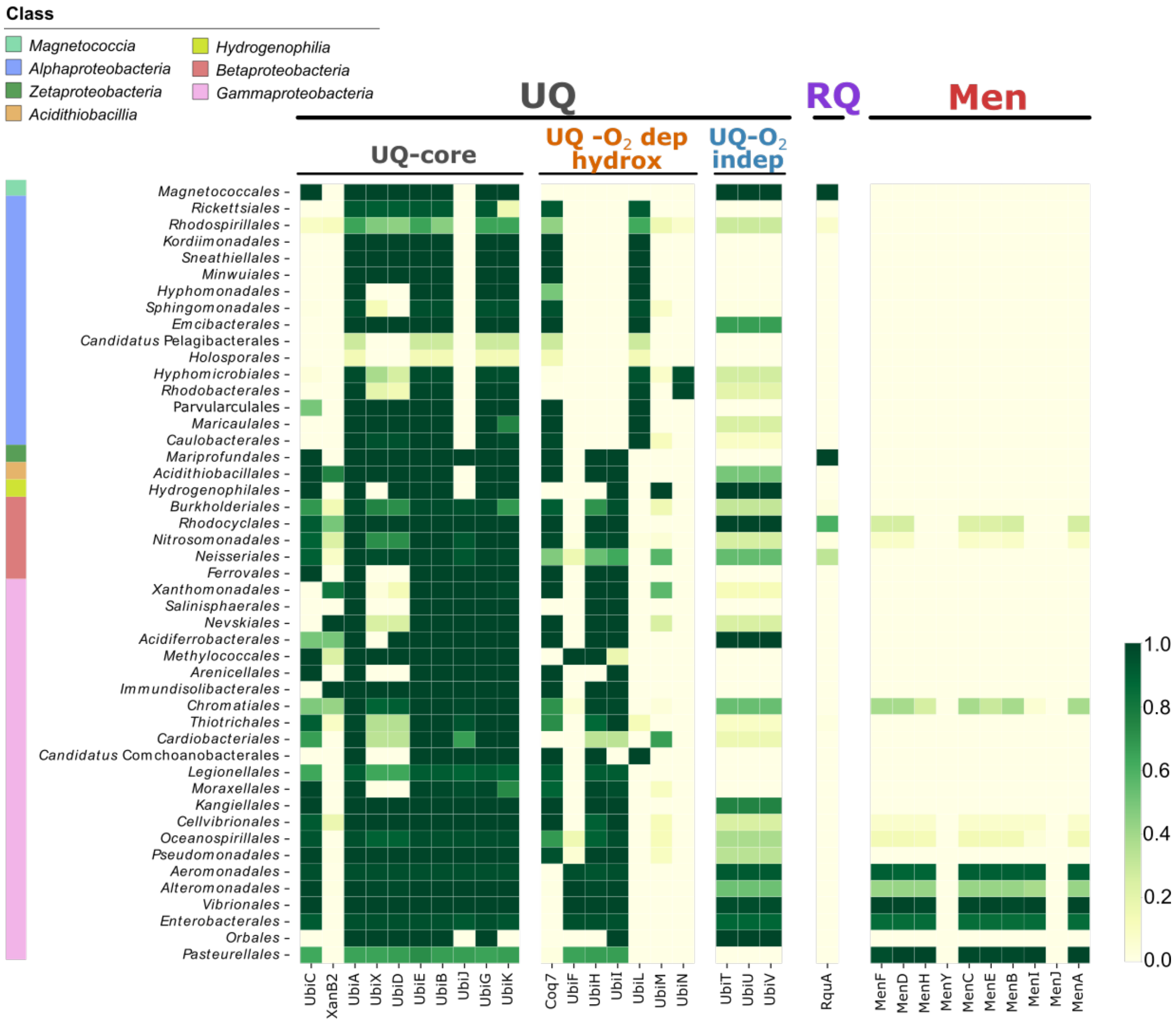

Fig. S12 Presence of quinone pathways genes at the order level in *Pseudomonadota*. The class corresponding to each order is depicted with a colored stripe on the left. Genes are separated by quinone type (UQ, RQ or MK) and by pathway. Genes are ordered according to their known or assumed position in their biosynthetic pathway (Fig. S1). The proportion of genomes with each gene is specified by taxonomic order with a green gradient.

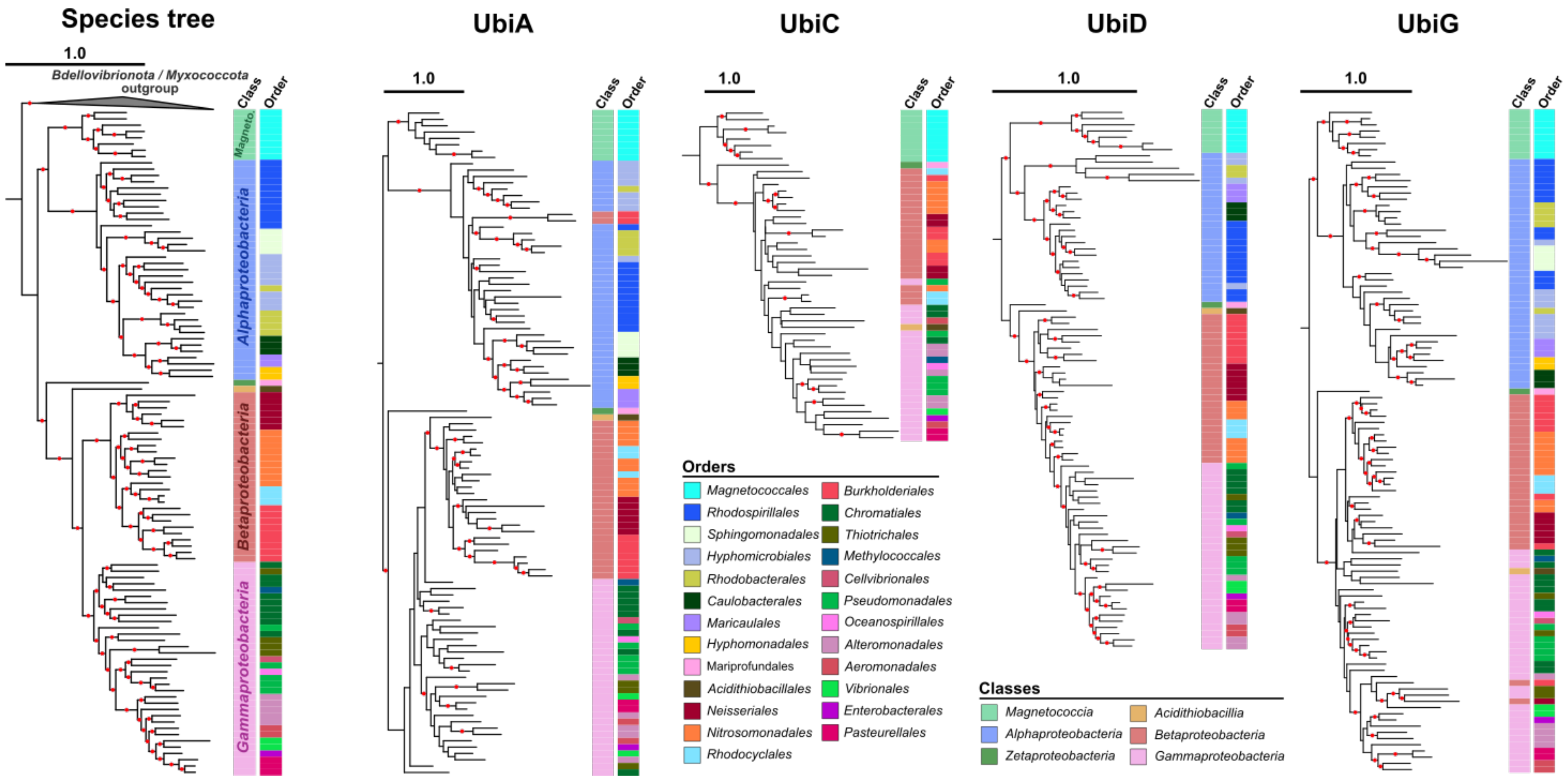

Fig. S13 Phylogenies of core genes of UQ pathways. The species tree is described in main Fig 3. The other trees are phylogenies of UQ core proteins: UbiA, -C, -D and -G. They are rooted such that they follow the species tree. The sequences come from species used to build the species tree. Every tree displays the class- and order-level taxonomy. They were obtained from the analysis of 257, 100, 482 and 208 aligned positions respectively using IQ-TREE with LG+F+R7 selected as the best model for UbiA, Q.pfam+I+G4 for UbiC, LG+I+I+R5 for UbiD, and Q.pfam+I+I+R6 for UbiG. The tree scale bars are expressed in number of substitutions per site. The branches with high support are indicated by red dots (UFBoot ≥ 90% for gene trees, UFBoot ≥ 95% for the species tree).

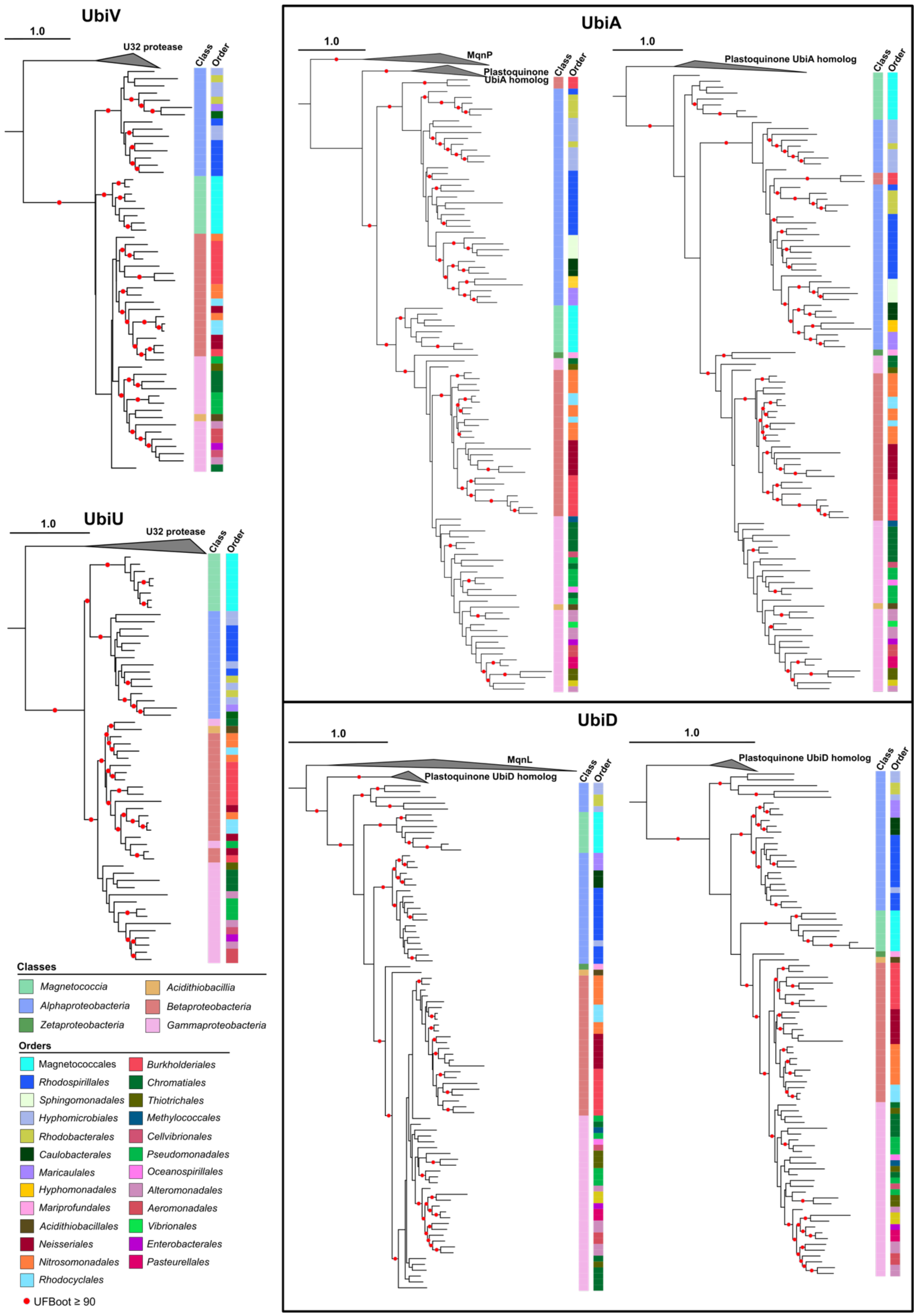

Fig. S14 Rooted phylogenies of UQ proteins: UbiV, -U, -A and -D. The UbiU and UbiV trees are rooted with sequences of U32 proteases, a large protein family they belong to. The sequences were taken from [3]. The UbiV tree was obtained from 255 aligned positions with LG+F+I+G4 selected as the best model. The UbiU tree was obtained from 303 aligned positions with Q.pfam+I+G4 selected as the best model. The UbiA and UbiD

trees were rooted by homologs from the plastoquinone biosynthesis pathway (corresponding to Slr0926 and Sll0936 respectively in *Synechocystis sp.* PCC 6803) and the MK futalosine pathway (MpnP for UbiA and MqnL for UbiD). They were both rooted with and without MqnP/MqnL, as the futalosine pathway homologs are the most distant. UbiA trees were made from 253 (with MqnP) and 252 (without MqnP) aligned positions, with respectively LG+F+R7 and LG+F+I+I+R7 selected as the best models. UbiD trees were made with 478 (with MqnL) and 479 (without MqnL) aligned positions, with respectively LG+I+I+R5 and LG+R6 selected as the best models. Phylogenies are congruent despite a lack of resolution in deep nodes. The *Magnetococcia* clade might be difficult to position in the trees, as demonstrated from the instability of its position in the UbiA and UbiD trees when changing the outgroup composition. However, the major clades (*Alpha-, Beta-, Gamma -proteobacteria*, and *Magnetococcia*) appear to be monophyletic, and the gene phylogenies globally reflected that of the species phylogeny presented in Fig. 3 for the UQ clade. All phylogenies were performed with IQ-TREE (see Materials and Methods). Every tree displays the class- and order-level taxonomy. Tree scale bars express the number of substitutions per site.

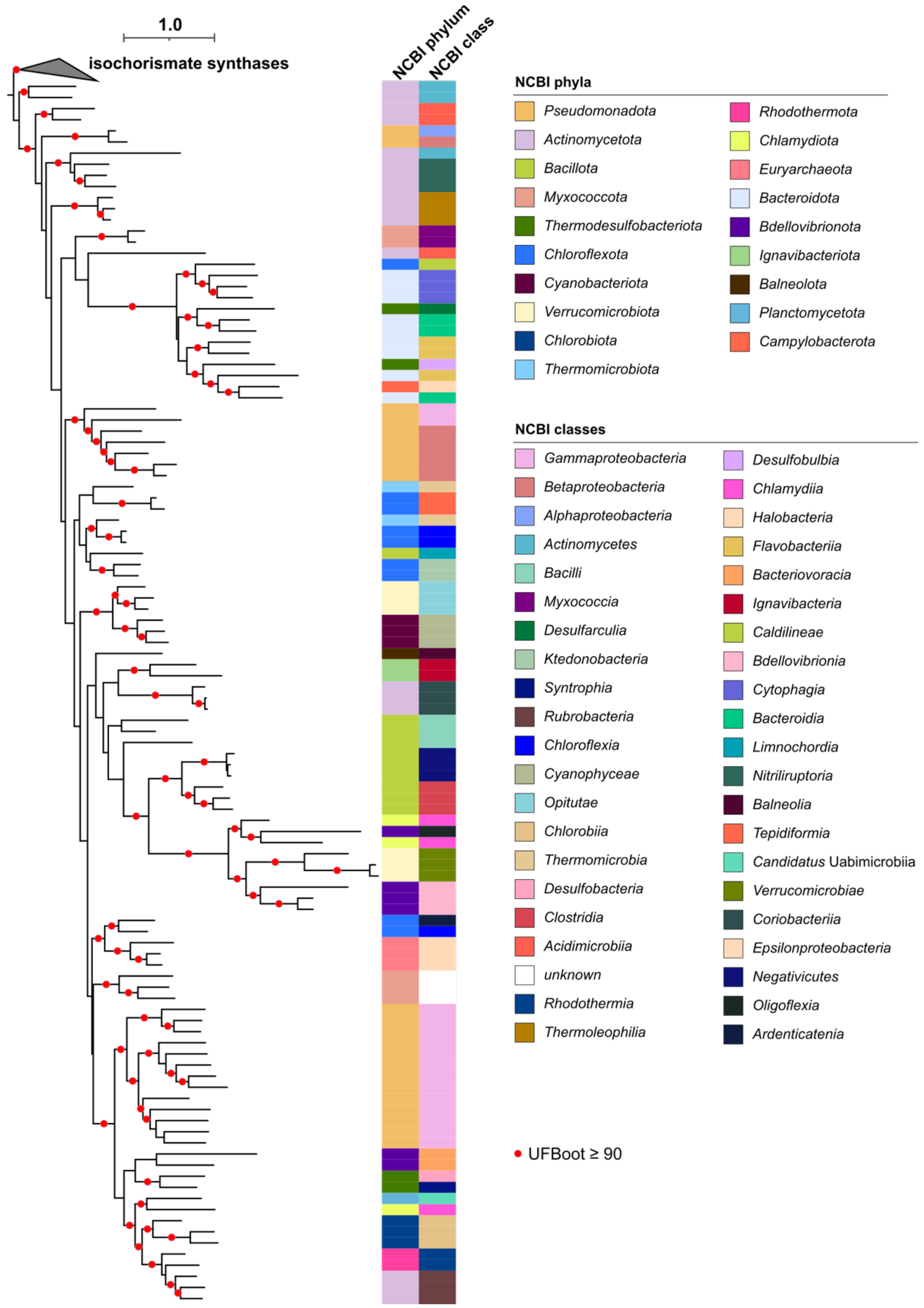

Fig. S15 Phylogeny of MenF. A maximum likelihood phylogeny of MenF is presented and was rooted with other isochorismate synthases sequences as an outgroup. The tree is annotated with color strips at two taxonomic levels: class and phylum. The branches with high support (UFBoot ≥ 90%) are indicated by red dots. The tree was obtained from the analysis of 217 aligned positions using IQ-TREE with LG+F+R7 as the best selected model. The tree scale bar expresses the number of substitutions per site.

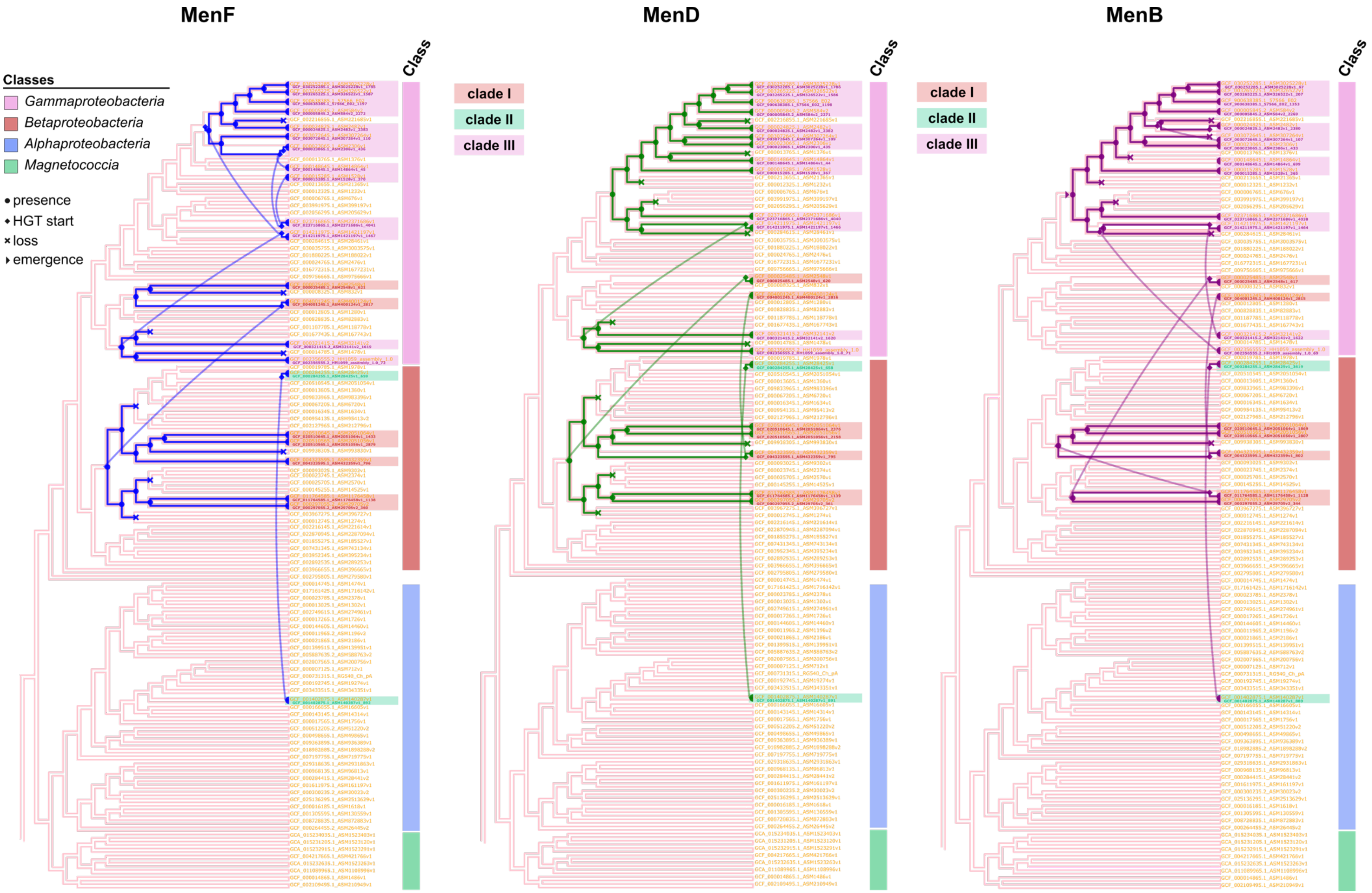

Fig. S16 Automatic reconciliation of the MenF, MenD and MenB phylogenies with the *Pseudomonadota* species tree. The species tree (in pink) corresponds to the species tree shown in Fig. 3, enriched with prokaryotic species with the Men pathway that were sampled to build the MenF, MenD, and MenB phylogenies (Fig. 4). The

construction of the species tree was performed using the same method as the tree in Fig. 3. Reconciliation was performed using GeneRax [4] and visualised with Thirdkind [5]. Here is displayed the part of the reconciliation corresponding to the Pseudomonadota. The inferred presence of a gene in a common ancestor is represented by a circle, a gene loss by a cross, a gene gain (emergence) by a triangle and horizontal gene transfer (HGT) by a diamond followed by a dashed arrow. The visualisation being focused on *Pseudomonadota*, the arrows showing HGT events coming from other prokaryotes are not displayed. We assume that the three genes underwent the same HGT events since they are located next to each other in genomes (except for one MenB, Fig. 4). Some events are likely to be artifacts from the reconciliation, such as the emergence of MenB in *Gammaproteobacteria*, certain losses, or mutiple HGT within *Gammaproteobacteria* in the MenF tree instead of a common origin as seen in the MenD and MenB trees. Artifacts appear to be most likely due to incorrect, unsupported gene trees. To infer the HGTs in Fig. 4, we chose the majority consensus (2/3) of the events inferred from the reconciliations of the three gene trees.

Reference

1. Joshi S, Fedoseyenko D, Mahanta N, Manion H, Naseem S, Dairi T, et al. Novel enzymology in futalosine-dependent menaquinone biosynthesis. *Curr Opin Chem Biol* 2018; **47**: 134–141.
2. Kazemzadeh K, Pelosi L, Chenal C, Chobert S-C, Hajj Chehade M, Jullien M, et al. Diversification of Ubiquinone Biosynthesis via Gene Duplications, Transfers, Losses, and Parallel Evolution. *Mol Biol Evol* 2023; **40**: msad219.
3. Kimura S, Sakai Y, Ishiguro K, Suzuki T. Biogenesis and iron-dependency of ribosomal RNA hydroxylation. *Nucleic Acids Res* 2017; **45**: 12974–12986.
4. Morel B, Kozlov AM, Stamatakis A, Szöllősi GJ. GeneRax: A Tool for Species-Tree-Aware Maximum Likelihood-Based Gene Family Tree Inference under Gene Duplication, Transfer, and Loss. *Mol Biol Evol* 2020; **37**: 2763–2774.
5. Penel S, Menet H, Tricou T, Daubin V, Tannier E. Thirdkind: displaying phylogenetic encounters beyond 2-level reconciliation. *Bioinformatics* 2022; **38**: 2350–2352.
