# Supplementary material for: Dynamic quinone repertoire accompanied the diversification of energy metabolism in Pseudomonadota
Source: ISME J. 2024 Dec 18;19(1):wrae253. doi: 10.1093/ismejo/wrae253 (PMC11707229; doi:10.1093/ismejo/wrae253)
Supplement: Sup_Texts_revised_wrae253 [file sup_texts_revised_wrae253.pdf]

Supplementary Information for:

Dynamic quinone repertoire accompanied the diversification of energy metabolism in Pseudomonadota

Sophie-Carole Chobert, Morgane Roger-Margueritat, Laura Flandrin, Safa Berraies, Christopher T. Lefèvre, Ludovic Pelosi, Ivan Junier, Nelle Varoquaux, Fabien Pierrel, Sophie S. Abby

Supplementary texts 3
- Text S1. Brief description of the quinone pathways to be annotated 3
- Text S2. Conservation of RquA function in RQ production across Pseudomonadota 3
- Text S3. Annotation of Quinone pathways in Pseudomonadota 3
- Text S4. Details on the distribution of the quinone biosynthetic enzymes within Pseudomonadota 4
- Text S5. Genetic organization and regulation of the *ubiTUV* loci 5

Supplementary methods 5
- Method S1. Heterologous expression of RquA proteins and activity measurements 5
- Method S2. Building HMM profiles for quinone biosynthetic proteins 6
- Methods S3. Permutation test for assessing the association between quinone pathways and metabolism type 7

References 8

Supplementary Datasets and Tables in separate files:

Dataset S1 – HMM profiles (online item, DOI: 10.6084/m9.figshare.26390974)
Dataset S2 – Optimized sequences of *rquA*
Dataset S3 – Annotated phylogenetic trees
Dataset S4 – Multiple Sequence Alignment and Phylogenetic trees (online item, DOI: 10.6084/m9.figshare.26391025)

Table S1 – Quinone genes, quinone pathways and taxonomy (NCBI/GTDB) of annotated genomes
- S1A – Annotated pathways in *Pseudomonadota*
- S1B – Annotated pathways in other prokaryotes
- S1C – Annotated proteins in *Pseudomonodata*
- S1D – Annotated proteins in other prokaryotes
- S1E – Pseudomonadota with no annotated quinone pathway

Table S2 – RquA experimental results
Table S3 – Genetic context of *ubiTUV*
Table S4 – FNR motifs discovery
- S4A – Discovered motifs
- S4B – *ubiTUV* triplets and FNR motifs

Table S5 – Quinone genes and quinone pathways in *Magnetococcia* and *Zetaproteobacteria*
- S5A – CheckM2 results
- S5B – *Magnetococcia* (HQ)
- S5C – *Zetaproteobacteria* (HQ)
- S5D – Annotated proteins in *Magnetococcia*
- S5E – Annotated proteins *Zetaproteobacteria*

Table S6 - Reductases in *Pseudomonadota*

S6A – Annotated HCO
S6B – Annotated cytochrome *bd*
Table S7 - Metabolism data for *Pseudomonadota*

Supplementary texts

Text S1. Brief description of the quinone pathways to be annotated

Two pathways are known for MK biosynthesis: the futalosine and the Men pathways [1] (Fig. S1). They each proceed in ~9 enzymatic steps from a chorismate precursor, with the addition of an isoprenoid chain at the antepenultimate step. 21 enzymes have been shown or proposed to be involved in the MK pathways [2, 3]. Demethyl-MK is the penultimate intermediate of these two pathways and is actually the end product when the MenG (also called UbiE) methyltransferase is missing, as is the case in some species [4]. UQ is produced in 9 steps from a chorismate precursor with the addition of the isoprenoid chain as a second step (Fig. S1). There are O_2-dependent and O_2-independent versions of the UQ pathway, which differ mainly in the enzymes involved in the three hydroxylation steps. In the O_2-independent pathway, which we recently discovered [5], three specific proteins (UbiT, UbiU, UbiV), including two hydroxylases, UbiU and UbiV, are involved in the hydroxylation steps and prephenate was identified as an oxygen donor [6]. In the O_2-dependent pathway, the oxygen donor is O_2, and we have recently unravelled the evolutionary and functional diversification of the corresponding hydroxylases, UbiF, -H, -I, -L, -M, -N and Coq7 [7, 8]. Rhodoquinone is produced from UQ in a single step via the RquA enzyme (Fig. S1), but the function of RquA has only been verified in *Rhodospirillum rubrum* [9, 10].

Text S2. Conservation of RquA function in RQ production across Pseudomonadota

Before inferring RQ production in our genomes dataset based on the sole presence of *rquA* and the UQ pathway, we first aimed at gaining confidence in the annotation of the *rquA* gene and the conservation of RquA function. Very few bacterial species have been described in the literature as RQ producers [11–13] and this scarcity is consistent with the sparse distribution for *rquA* observed in prokaryotes [14]. Our HMM profiles identified RquA proteins only in Pseudomonadota. With these sequences, we built a maximum likelihood phylogenetic tree using IQ-tree (Fig. S6A) and we used as an outgroup UbiE sequences, a methyltransferase from the same family as RquA [14]. We then sampled 16 sequences along the phylogeny of RquA to assess their function experimentally. To this end, we used heterologous expression in *E. coli* which produces UQ endogenously but not RQ. Expression of the RquA homologs from *P. photometricum* and *A. vannielii* in *E. coli* led to the production of a compound eluting at 6.8 min, which was established to be RQ_8 based on mass spectrometry analysis (Fig. S6B-D, Methods S1). Out of the 16 sequences tested, 15 allowed the production of RQ_8 (Fig. S6E-G, Table S2). These results demonstrate that the tested sequences have the same function as the RquA protein characterized in *R. rubrum*. Therefore, we can confidently annotate RQ biosynthesis in our genomic dataset based on the annotation of a UQ pathway plus a RquA protein.

Text S3. Annotation of Quinone pathways in Pseudomonadota

To determine the occurrence of quinones in *Pseudomonadota*, we annotated their biosynthetic pathways in complete genomes of 4107 species of Pseudomonadota as well as on a subset of 5392 genomes representative of the diversity of the prokaryotes as a benchmark dataset. To this end we used hidden Markov model (HMM) protein profiles for each protein known or proposed to participate in the production of UQ/RQ and (D)MK and set up minimal numbers of required proteins to establish the presence of the respective pathways (Figs. S1, S8). As expected [15], we retrieved very few hits for proteins of the futalosine pathway in Pseudomonadota [15], whereas genomes of other prokaryotes displayed many hits that formed a bimodal distribution when plotting the number of different proteins

found in genomes (Fig. S8). A minimum number of proteins required to infer the presence of the pathway was set at the beginning of the second mode (6 proteins), above which we consider that the futalosine pathway is present in a given species. Similarly, we set a threshold of 6 proteins for the Men pathway (Fig. S8). Overall, we inferred MK biosynthesis in 891 species of *Pseudomonadota*, exclusively via the Men pathway (Table S1, Text S2).

The O_2-dependent and O_2-independent UQ pathways share a common part composed of up to 9 proteins (UbiC, XanB2, UbiA, UbiB, UbiX, UbiD, UbiB, UbiE and UbiG) and possess additionally either O_2-dependent hydroxylases (combinations of UbiH, UbiI, UbiF, UbiM, UbiL, UbiN, Coq7 [7]) or the UbiT, -U, -V proteins [5], respectively (Fig. 1). UbiT, -U, -V were almost always detected together and exclusively in genomes of *Pseudomonadota* (Fig. S8). We retrieved very few hits for UQ O_2-dependent hydroxylases outside of *Pseudomonadota* and found from 0 to 4 proteins in genomes of *Pseudomonadota* (Fig. S8), in agreement with our previous results [7]. Overall, we infer the presence of the UQ O_2-dependent pathway based on at least 4 proteins in the common part and at least one O_2-dependent hydroxylase. We predict the presence of the UQ O_2-independent pathway based on at least 4 proteins in the common part and hits for the three proteins UbiT, -U, -V. Applying these rules, we found UQ pathways exclusively in *Pseudomonadota* when searching bacterial genomes (Fig. S8, Table S1). We inferred the UQ O_2-dependent pathway in 4009 species (97.6%) and the UQ O_2-independent pathway in 1562 species (39%). Moreover, our results show that the protein composition of the common part of the UQ pathway is quite variable (Fig. S12).

Text S4. Details on the distribution of the quinone biosynthetic enzymes within Pseudomonadota

Based on comparative genomics, MenI and MenY were previously proposed to be part of the Men pathway [3], but we found no support for this hypothesis in *Pseudomonadota* (Figs. S4-5, S12). Our annotation of the UQ pathway across *Pseudomonadota* revealed a core of four conserved proteins (UbiA, UbiB, UbiG, and UbiE) in all genomes annotated as producing UQ and a variable distribution for other proteins (Fig. S12). In addition to the diversity of O_2-dependent hydroxylases that we recently documented [7], we noted the patchy distribution of the decarboxylase UbiD and its associated protein UbiX, both of which are largely absent from several orders of *Alpha-, Beta-, and Gamma- proteobacteria* (Fig. S12, Table S1). This suggests that the decarboxylation step may be performed by other proteins in these lineages. One potential candidate is a recently discovered flavin monooxygenase that catalyzes an oxidative decarboxylation in *Rhodobacter capsulatus* and replaces UbiX and UbiD under aerobic conditions [16]. Whether this protein is conserved in other Pseudomonadota remains to be investigated. Alphaproteobacteria have the most reduced UQ pathway (Fig. S12, Table S1), as they generally lack UbiC, which is involved in the production of the 4-hydroxybenzoate precursor from chorismate (Fig. S1) and they completely lack UbiJ, which structures the UQ biosynthetic complex in *E. coli* [17]. UbiT from the O_2-independent UQ pathway is a UbiJ homolog but is not sufficiently conserved across *Alphaproteobacteria* to functionally replace UbiJ (Fig. S12). Taken together, the missing steps and the extensive diversification of the UQ pathway suggest that UQ biosynthetic enzymes remain to be discovered. A comparison of UQ-producing proteins in mitochondria and Alphaproteobacteria may provide insights on the missing enzymes in the *Pseudomonadota* pathway and on the origin of UQ in eukaryotes.

Text S5. Genetic organization and regulation of the *ubiTUV* loci

The genetic organization of *ubiTUV* is highly conserved because *ubiV* always follows *ubiU*, and *ubiT* is located either upstream or downstream of the conserved *ubiUV* pair on the same strand or on the complementary strand (Fig. 2B, Table S3). Therefore, four main genetic architectures of *ubiTUV* (out of 18 possible) are found across the Pseudomonadota and their distribution follows the phylogeny (Fig. 2A). Architectures "1" and "2" with *ubiT* either preceding or following *ubiUV* on the same strand, are largely represented in Alphaproteobacteria (97%) and Betaproteobacteria (90%), respectively. Gammaproteobacterial genomes exhibit mainly architecture "3" with the order *ubiT*, *-U* and *-V*, with *ubiT* on the opposite strand (63%). Architecture "4" corresponds to *ubiUV* at one locus, and *ubiT* at least 500 bp away (Fig. 2A-B, Table S3). Architecture "4" is found in 21% and 7% of genomes from *Gammaproteobacteria* and *Betaproteobacteria*, which consist mostly of *Vibrionales*, and *Burkholderiales*, respectively. The strong conservation of the *ubiTUV* genes at a single locus suggests a co-regulation process. Therefore, we investigated the sequences upstream of the *ubiTUV* genes and identified FNR-binding sites as the most significant potential regulatory sites.

Whenever *ubiTUV* are on the same strand (architectures "1" and "2"), FNR-binding sites are predicted mostly before the first gene of the triplet with frequencies of 78% and 65% for *ubiU* and *ubiT,* respectively (Fig. 2B). With architecture "3" where *ubiT* and *ubiUV* are on opposite strands and directions, 96% of the cases show a predicted FNR site between *ubiU* and *ubiT*. Regarding architecture "4" where *ubiT* is found apart from *ubiUV*, the candidate sites tend to be located before *ubiU* in 74% of the cases and/or *ubiT* in 64% of the cases (Fig. 2B). When considering the position of the candidate sites relative to the start codon, we found a median position between -69 and -72 nucleotides (nt) for architectures "1", "2" and "4" (Figs. 2B, S5A-B, S5D). As for the position of the candidate FNR sites in the architecture "3" that predominates among *Gammaproteobacteria*, the median of their distribution is -87 nt before *ubiU* and -130 nt before *ubiT* (Figs. 2B, S5C). In *E. coli*, when FNR acts as a positive regulator, its binding site is usually centered at -40.5 bp from the transcription start site (TSS) of the regulated gene [18]. The center of the FNR binding site controlling *ubiU* is located at -43.5 bp from the TSS [19], which corresponds to a binding site centered at -87.5 from the start codon. This is fully consistent with the -87 median position found upstream *ubiU* in *Gammaproteobacteria* (Fig. 2B). The role of FNR or FNR homologs in the expression of the UQ O_2-independent pathway has been formally demonstrated only in *E. coli* [20] and *P. aeruginosa* [18] and is highly suggested from expression data in the alphaproteobacterium *Sphingopyxis granuli* [21]. Overall, our results support a conserved role of FNR in the joint positive regulation of *ubiT, -U, -V* throughout Pseudomonadota, resulting in the activation of the UQ O_2-independent pathway when O_2 levels decrease.

Supplementary methods

Method S1. Heterologous expression of RquA proteins and activity measurements

The RquA proteins experimentally tested in this study are listed in Table S2. The nucleotide sequences were optimized for expression in *E. coli* and are available in Dataset S1. The genes were synthesized by the "Genecust" company and cloned into the pBAD24i vector downstream of arabinose-inducible promoter using NcoI (5' end) and HindIII (3' end) restriction enzymes. The ATG start codon of the

rquA genes is comprised in the NcoI site sequence (CCATGG), which imposes a G as the first nucleotide of the second codon. In case the second amino acid did not correspond to a codon starting with a G, an alanine codon (GCG) was added before the second codon of the nucleotide sequence, as in [7].

The *E. coli* MG1655 strain was transformed by the pBAD24i vectors carrying the *rquA* genes and selected on lysogeny broth (LB) plates supplemented with ampicillin (100 µg/mL). Individual clones were inoculated in 1.5 mL LB medium with ampicillin (100 µg/mL) and grown overnight at 37°C in closed Eppendorf tubes. These pre-cultures were used to inoculate, at OD_{600} ~ 0.02, cultures of 5 mL LB medium containing 0.05% (wt/vol) arabinose and ampicillin (100 µg/mL). The aerobic cultures were grown overnight in 50 mL Erlenmeyer flasks at 37°C with 180 rpm shaking. The 5 mL cultures were cooled on ice for 30 min before centrifugation at 3200 x g at 4°C for 10 min. Cell pellets were washed in 1 mL ice-cold phosphate-buffer saline (PBS) and transferred to pre-weighted 1.5 mL Eppendorf tubes. After centrifugation at 12,000 g at 4 °C for 1 min, the supernatant was discarded, the cell wet weight was determined and pellets were stored at -20°C until lipid extraction, if necessary. The activity of the various RquA proteins was evaluated by measuring RQ levels in lipid extracts of the cells.

Quinone extraction from cell pellets and analysis by HPLC-ECD-MS were performed as previously described [5]. MS detection was performed with a MSQ spectrometer (Thermo Scientific) with electrospray ionization in positive mode (probe temperature, 400°C; cone voltage, 80 V). Single-ion monitoring detected the following compounds: UQ_8 ($M+NH_4^+$), m/z 744-745, 6–10 min, scan time of 0.2 s; UQ_{10} ($M+NH_4^+$), m/z 880–881, 10-17 min, scan time of 0.2 s; RQ_8 ($M+H^+$), m/z 712–713, 4–12 min, scan time of 0.2 s. MS spectra were recorded between m/z 600 and 1000 with a scan time of 0.3 s. The area of the MS peaks for RQ_8 were corrected for sample loss during extraction on the basis of the recovery of the UQ_{10} internal standard and were normalized to cell wet weight and to the slope of the MS calibration curve of UQ_{10}.

Method S2. Building HMM profiles for quinone biosynthetic proteins

To identify proteins from the MK (men and futalosine) pathway, O_2-dependent and O_2-independent UQ pathways and the RQ biosynthetic pathway, hidden Markov model (HMM) profiles were used (Fig. 1). We employed previously published HMM profiles for UbiA, MenA, UbiE (MenG), UbiT, UbiU, UbiV, U32 proteases, Coq7, UbiF, UbiH, UbiI, UbiL, UbiN, UbiM and flavin monooxygenases (FMO) [5, 7, 22]. In addition, we designed HMM protein profiles for the remaining UQ enzymes and the two MK pathways following the same approach as before. Briefly, we took an approach starting from reference sequences for the enzymes, and iterations of i) similarity searches and sequence clustering in genomes, ii) phylogenetic analyses with IQ-TREE [23] (see below) and sequence selection for HMM profiles design, iii) curation of the HMM profiles using phylogenetic analyses and, in some cases, the genomic context of the genes. This was iterated until HMM profiles were deemed sensitive and specific enough. In some cases, we built decoy HMM profiles to sort out the homologs with different functions and GA (gathering) bit score thresholds were set for the HMM profiles lacking specificity such as for MenA, MenB, MenE and MenH. We created two RquA profiles from the sequences retrieved in [14]. We built a phylogenetic tree with the sequences annotated as RquA in our dataset and that of Stairs and colleagues and deduced from it a cut GA score for each profile. In the end, a total of 95 HMM protein profiles were obtained (including those published) with the *hmmbuild* command of HMMER v3.3.2 [24] from multiple sequence alignments obtained with MAFFT (l-ins-i) trimmed at their extremities using BMGE (BLOSUM 30 matrix used) [25, 26]. These corresponded to the annotation of UbiA, -B, -C, -D, -E, -F, -G, -H, -I, -L, -M, -N, -T, -U, -V, and XanB2 from the UQ pathway, RquA from the RQ pathway, MenA, -B, -C, -D, -E, -F, -G, -H, -I, -Y

from the Men MK pathway, and MqnA, -B, -C, -D, -E, -L, -M, -P, -Z, and MtnN from the futalosine MK pathway.

Methods S3. Permutation test for assessing the association between quinone pathways and metabolism type

In order to assess the association between quinone pathways and metabolism type, we developed a permutation test. We categorize organisms in four categories: ones with presence of the UQ O_2-dependent pathway (category A), ones with both UQ – O_2-dependent and O_2-independent pathways (category B), ones with both HP and LP quinone pathways (category C), and ones with only LP quinone pathways (category D). To assess whether the proportion of aerobic organisms decreases along these ordered categories:

1. Compute the percentage of aerobic organisms in each of the categories $(\%A, \%B, \%C, \%D)$ and the statistic:

   $$S = min\ (\%A - \%B, \%B - \%C, \%C - \%D)\,. \qquad (1)$$

   A larger statistic S indicates that $\%A$ is much greater than $\%B$, $\%B$ is much greater than $\%C$, and $\%C$ is much greater than $\%D$. A negative statistic indicates that the percentage of organisms with the trait of interest in category A is smaller in category B, B versus C, or C versus D.

2. For $b = 1, \dots, B$, where B is a large integer (e.g., 10000):

   (a) Permutate the metabolism annotation (aerobic, facultative, anaerobe) for each genome.
   (b) Compute the statistics Sb on the permutated genome.

3. The *P* value is given by:

   $$\frac{1}{B+1} \sum_{b=1}^{B} \left(1_{(S^b >= S)} + 1\right), \qquad (2)$$

where $1_{S^b \geq S}$ is an indicator variable that is equal to 1 if $S^b \geq S$, and 0 otherwise. Note that the *P* value is never 0 [27].

References

1. Hiratsuka T, Furihata K, Ishikawa J, Yamashita H, Itoh N, Seto H, et al. An alternative menaquinone biosynthetic pathway operating in microorganisms. *Science* 2008; **321**: 1670–1673.
2. Kawamukai M. Biosynthesis and applications of prenylquinones. *Biosci Biotechnol Biochem* 2018; **82**: 963–977.
3. Ravcheev DA, Thiele I. Genomic analysis of the human gut microbiome suggests novel enzymes involved in quinone biosynthesis. *Front Microbiol* 2016; **7**: 128.
4. Rosenberg E, DeLong EF, Lory S, Stackebrandt E, Thompson F (eds). The Prokaryotes: *Gammaproteobacteria*. 2014. Springer Berlin Heidelberg, Berlin, Heidelberg.
5. Pelosi L, Vo C-D-T, Abby SS, Loiseau L, Rascalou B, Hajj Chehade M, et al. Ubiquinone biosynthesis over the entire O_2 range: characterization of a conserved O_2-independent pathway. *mBio* 2019; **10**: e01319-19.
6. Kazemzadeh Ferizhendi KK, Simon P, Pelosi L, Séchet E, Arulanandam R, Chehade MH, et al. An organic O donor for biological hydroxylation reactions. *Proc Natl Acad Sci USA* 2024; **121**: e2321242121.
7. Kazemzadeh K, Pelosi L, Chenal C, Chobert S-C, Hajj Chehade M, Jullien M, et al. Diversification of ubiquinone biosynthesis *via* gene duplications, transfers, losses, and parallel evolution. *Mol Biol Evol* 2023; **40**: msad219.
8. Pelosi L, Ducluzeau A-L, Loiseau L, Barras F, Schneider D, Junier I, et al. Evolution of ubiquinone biosynthesis: multiple proteobacterial enzymes with various regioselectivities to catalyze three contiguous aromatic hydroxylation reactions. *mSystems* 2016; **1**.
9. Lonjers ZT, Dickson EL, Chu T-PT, Kreutz JE, Neacsu FA, Anders KR, et al. Identification of a new gene required for the biosynthesis of rhodoquinone in *Rhodospirillum rubrum*. *J Bacteriol* 2012; **194**: 965–971.
10. Neupane T, Chambers LR, Godfrey AJ, Monlux MM, Jacobs EJ, Whitworth S, et al. Microbial rhodoquinone biosynthesis proceeds via an atypical RquA-catalyzed amino transfer from S-adenosyl-L-methionine to ubiquinone. *Commun Chem* 2022; **5**: 1–13.
11. Hiraishi A. Fumarate reduction systems in members of the family *Rhodospirillaceae* with different quinone types. *Arch Microbiol* 1988; **150**: 56–60.
12. Hiraishi A, Hoshino Y. Distribution of rhodoquinone in *Rhodospirillaceae* and its taxonomic implications. *J Gen Appl Microbiol* 1984; **30**: 435–448.
13. Miyadera H, Hiraishi A, Miyoshi H, Sakamoto K, Mineki R, Murayama K, et al. Complex II from phototrophic purple bacterium *Rhodoferax fermentans* displays rhodoquinol-fumarate reductase activity. *Eur J Biochem* 2003; **270**: 1863–1874.
14. Stairs CW, Eme L, Muñoz-Gómez SA, Cohen A, Dellaire G, Shepherd JN, et al. Microbial eukaryotes have adapted to hypoxia by horizontal acquisitions of a gene involved in rhodoquinone biosynthesis. *eLife* 2018; **7**: e34292.
15. Zhi X-Y, Yao J-C, Tang S-K, Huang Y, Li H-W, Li W-J. The futalosine pathway played an important role in menaquinone biosynthesis during early prokaryote evolution. *Genome Biol Evol* 2014; **6**: 149–160.
16. Nagatani H, Mae Y, Konishi M, Matsuzaki M, Kita K, Daldal F, et al. UbiN, a novel *Rhodobacter capsulatus* decarboxylative hydroxylase involved in aerobic ubiquinone biosynthesis. *FEBS Open Bio* 2023; **13**: 2081–2093.
17. Hajj Chehade M, Pelosi L, Fyfe CD, Loiseau L, Rascalou B, Brugière S, et al. A soluble metabolon synthesizes the isoprenoid lipid ubiquinone. *Cell Chem Biol* 2019; **26**: 482-492.e7.
18. Myers KS, Yan H, Ong IM, Chung D, Liang K, Tran F, et al. Genome-scale analysis of *Escherichia coli* FNR reveals complex features of transcription factor binding. *PLoS Genet* 2013; **9**: e1003565.
19. Arias-Cartin R, Kazemzadeh Ferizhendi K, Séchet E, Pelosi L, Loeuillet C, Pierrel F, et al. Role of the *Escherichia coli* ubiquinone-synthesizing UbiUVT pathway in adaptation to changing respiratory conditions. *mBio* 2023; **14**: e03298-22.
20. Kroppenstedt RM, Mannheim W. Lipoquinones in members of the family *Pasteurellaceae*. *Int J Syst Bacteriol* 1989; **39**: 304–308.

21. González-Flores YE, de Dios R, Reyes-Ramírez F, Santero E. Identification of two fnr genes and characterisation of their role in the anaerobic switch in *Sphingopyxis granuli* strain TFA. *Sci Rep* 2020; **10**: 21019.
22. Launay R, Teppa E, Martins C, Abby SS, Pierrel F, André I, et al. Towards molecular understanding of the functional role of UbiJ-UbiK2 complex in ubiquinone biosynthesis by multiscale molecular modelling studies. *Int J Mol Sci* 2022; **23**: 10323.
23. Minh BQ, Schmidt HA, Chernomor O, Schrempf D, Woodhams MD, von Haeseler A, et al. IQ-TREE 2: new models and efficient methods for phylogenetic inference in the genomic era. *Mol Biol Evol* 2020; **37**: 1530–1534.
24. Eddy SR. Accelerated profile HMM searches. *PLOS Comp Biol* 2011; **7**: e1002195.
25. Katoh K, Standley DM. MAFFT: iterative refinement and additional methods. *Methods Mol Biol* 2014; **1079**: 131–146.
26. Criscuolo A, Gribaldo S. BMGE (Block Mapping and Gathering with Entropy): a new software for selection of phylogenetic informative regions from multiple sequence alignments. *BMC Evol Biol* 2010; **10**: 210.
27. Phipson B, Smyth GK. Permutation P-values should never be zero: calculating exact P-values when permutations are randomly drawn. *Stat Appl Genet Mol Biol* 2010; **9**.
